# Drivers of Bushmeat Hunting and Perceptions of Zoonoses in Nigerian Hunting Communities

**DOI:** 10.1371/journal.pntd.0003792

**Published:** 2015-05-22

**Authors:** Sagan Friant, Sarah B. Paige, Tony L. Goldberg

**Affiliations:** 1 Nelson Institute for Environmental Studies, University of Wisconsin, Madison, Wisconsin, United States of America; 2 Department of Pathobiological Sciences, University of Wisconsin, Madison, Wisconsin, United States of America; 3 Global Health Institute, University of Wisconsin, Madison, Wisconsin, United States of America; Tulane School of Public Health and Tropical Medicine, UNITED STATES

## Abstract

Bushmeat hunting threatens biodiversity and increases the risk of zoonotic pathogen transmission. Nevertheless, limited information exists on patterns of contact with wildlife in communities that practice bushmeat hunting, especially with respect to social drivers of hunting behavior. We used interview responses from hunters and non-hunters in rural hunting communities in Nigeria to: 1) quantify contact rates with wildlife, 2) identify specific hunting behaviors that increase frequency of contact, 3) identify socioeconomic factors that predispose individuals to hunt, and 4) measure perceptions of risk. Participants engaged in a variety of behaviors that increased contact with wild animals, including: butchering to sell (37%), being injured (14%), using body parts for traditional medicine (19%), collecting carcasses found in forests and/or farms (18%), and keeping as pets (16%). Hunters came into contact with wildlife significantly more than non-hunters, even through non-hunting exposure pathways. Participants reported hunting rodents (95%), ungulates (93%), carnivores (93%), primates (87%), and bats (42%), among other prey. Reported hunting frequencies within taxonomic groups of prey were different for different hunting behaviors. Young age, lower education level, larger household size, having a father who hunts, and cultural group were all associated with becoming a hunter. Fifty-five percent of respondents were aware that they could contract diseases from wild animals, but only 26% of these individuals reported taking protective measures. Overall, hunters in this setting frequently contact a diversity of prey in risky ways, and the decision to become a hunter stems from family tradition, modified by economic necessity. Conservation and public health interventions in such settings may be most efficient when they capitalize on local knowledge and target root socio-economic and cultural drivers that lead to hunting behavior. Importantly, interventions that target consumption alone will not be sufficient; other drivers and modes of interaction with wildlife must also be considered.

## Author Summary

Bushmeat hunting threatens biodiversity and increases the risk of disease transmission from animals to people. Using interview methods, we described patterns of hunting, potential pathways for zoonotic disease transmission, and drivers of hunting behavior in hunting communities in Southeast Nigeria. Participants contacted a diversity of wildlife through multiple pathways, but were not generally aware of wildlife zoonoses and typically took no or inadequate protective measures. Bushmeat was highly preferred over domestic alternatives, but there was a negative view of hunting as a livelihood, even among hunters themselves. Family size, age, education and family and cultural tradition influenced whether individuals hunted. Wildlife were hunted for many reasons other than consumption, indicating the importance of alternative pathways for zoonotic disease transmission. In this setting, sociocultural factors appear to be more important drivers of hunting behavior (and zoonotic disease risk) than previously appreciated. Our results will help inform policies aimed at modifying the impact of hunting on African forest wildlife and preventing zoonotic disease transmission.

## Introduction

An estimated 282 grams of bushmeat are consumed per person per day in the Congo Basin, with over three million tons harvested in Central Africa annually [1,2]. Hunting of wild animals on this scale threatens wildlife conservation and increases risk of zoonotic disease transmission [3,4]. Rural communities across the tropical forests of West and Central Africa rely heavily on bushmeat as a nutritional, economic and cultural component of their livelihoods [5,6]. However, increasingly intense extraction is unsustainable and results in enhanced opportunities for zoonotic disease transmission [7]. A general shift towards cash economies, increased access to previously remote areas for natural resource extraction, and widespread use of guns have altered traditional hunting behavior and increased dependency on the sale of bushmeat to meet urban demands [8–12]. Market surveys in Nigeria estimate that over 900,000 kilograms of bushmeat are sold annually [13]. Large profit margins create incentives for the bushmeat trade across all levels of the supply chain, allowing bushmeat to reach national and international markets [13]. In the Ivory Coast, for example, the bushmeat trade is valued at 150 million USD [2]. An estimated five tons of bushmeat are smuggled from Africa to Europe per week [14]. Worldwide, wildlife is second only to narcotics among black market trades [15].

Frequent contact with wildlife through the bushmeat trade puts people at risk of infection with zoonotic pathogens. Pathogens transmissible to humans through bushmeat include: simian immunodeficiency virus, human T-cell lymphotrophic virus, simian foamy virus, monkeypox virus, Ebola and Marburg filoviruses, anthrax, herpes viruses, hepatitis viruses, paramyxoviruses and various parasites [16]. Among prey taxa, bats, rodents and primates consistently stand out as important sources of zoonoses. Bats and rodents have high zoonotic viral richness, and the close genetic similarity between humans and non-human primates makes exposure particularly risky [17–20]. For example, pandemic HIV originated from viruses of Central African chimpanzees, providing a striking example of the global consequences of zoonoses resulting from contact with primates [21]; and other simian retroviruses appear to “jump” between primates and people with regularity (for review see: [22]). Compared to primates, rodents are a far more abundant and geographically widespread taxon [23,24]. Forest dwelling and peridomestic rodents in West Africa host viruses such as Lassa virus and monkeypox virus, as well as a range of vector-borne pathogens [18]. Bats harbor the highest number of zoonotic viruses per host species and have received a great deal of recent attention because of outbreaks of zoonotic corona-, filo-, and paramyxoviruses [25,26]. The nature and frequency of human interaction with these and other wildlife taxa determine the pathways by which zoonotic diseases emerge.

The disruption of transmission pathways requires improved understanding of the interactions between key biological, behavioral and sociological drivers of human—animal contact. In places where reliance on wild foods and income are linked, certain individuals may be at particular risk of infection. Conventional wisdom holds that the poorest households in rural communities rely most heavily on wild foods [27–31], but this paradigm is not universal [32–34]. Still, little information exists on social and economic factors that influence whether individuals hunt.

In this study, we conducted interviews in remote Nigerian hunting communities to identify: 1) transmission pathways by nature and frequency of interactions between humans and wildlife; and 2) socioeconomic factors that may put individuals at increased risk of zoonotic infections from wild animals. Because perceptions of risk are known to vary among hunters in West and Central Africa [30,35], we also used closed- and open-ended interviews to measure zoonotic disease awareness, perceived risk and self-protective behavior.

### Study Site

**Fig 1:**
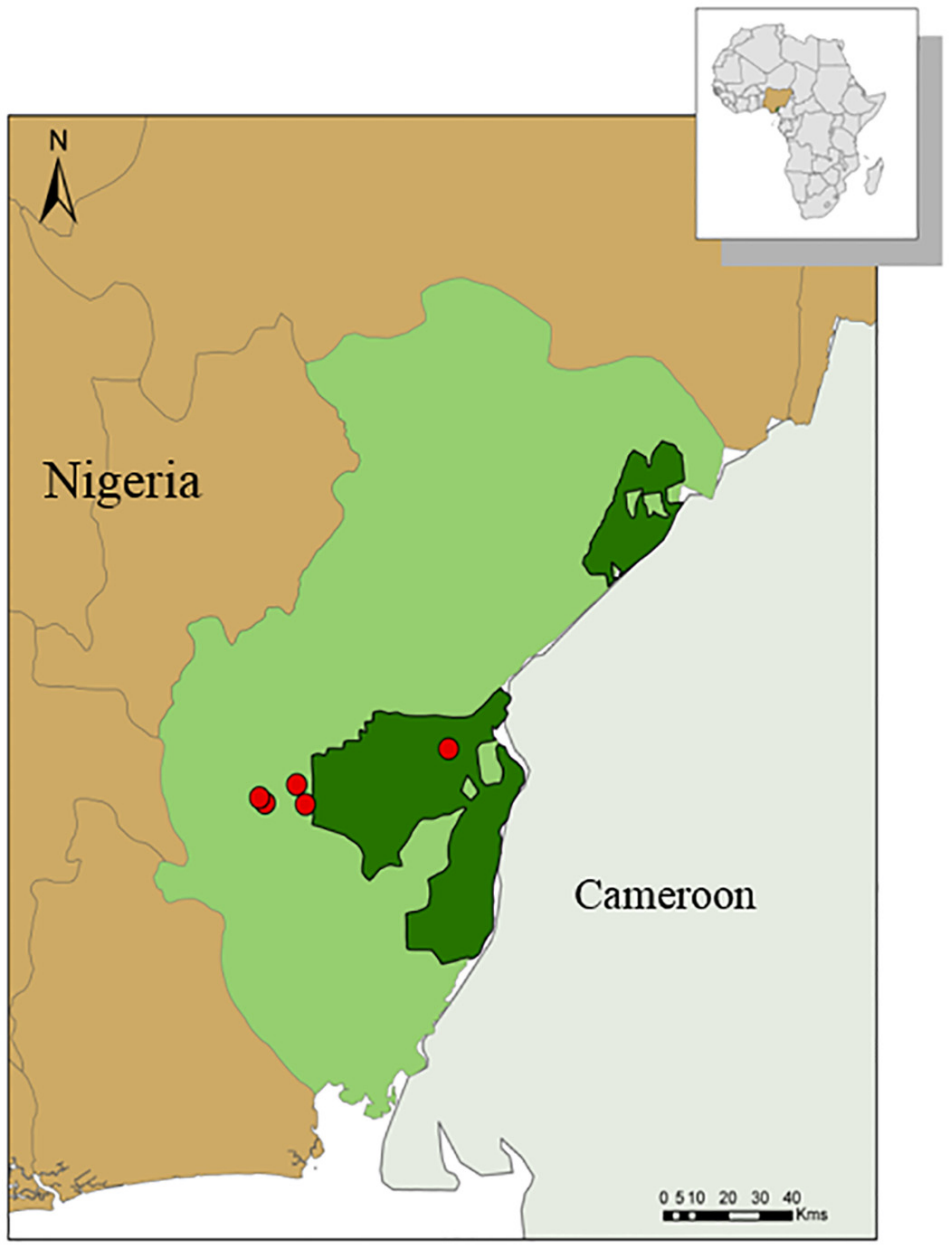
Study sites. Map showing location of study communities relative to the Oban Division of Cross River National Park (dark green) in Cross River State, Nigeria (light green).

We conducted interviews in five rural hunting communities near the Oban Division of Cross River National Park in Cross River State, Nigeria (Fig 1). The park was created in 1991 and has two non-contiguous divisions. The southern Oban division is about 3,000 km^2^ of lowland rainforest, making it the largest closed-canopy rainforest in Nigeria. It is ecologically contiguous to Korup National Park in Cameroon and is recognized as a biodiversity and infectious disease hotspot, where pathogen transmission from wildlife to humans is most likely [19,36,37]. Illegal logging, agricultural expansion and hunting threaten the park. The forest surrounding the Oban division is characteristic of lowland rainforest, forming a mosaic of disturbed and relatively undisturbed forest patches. To increase the generality of our results, we selected communities that varied in proximity to the national park (outside, support zone, or enclave) and cultural group (primarily Efik or Ejagham).

## Methods

### Study Design

We interviewed 327 participants between August and December 2012. All interviews were conducted in Nigerian Pidgin English, a language spoken as *lingua franca* across Nigeria, by the first author with the assistance of local translators when necessary. Administrative visits to each village preceded interviews to meet with clan heads, chief hunters and hunter groups, hold informational sessions and request permission for research activities.

We enrolled participants to obtain responses from an approximately equal number of hunters and non-hunters. We first enrolled self-identified hunters and then identified non-hunters through random-selection of households. If household members chose not to participate, or were not home after three visits, we replaced the household with its nearest neighbor. During non-hunter interviews, we frequently discovered individuals who were, in fact, actively hunting or had hunted previously in their lives, and in several villages, we were unable to find a sufficient number of people who had never hunted. Because we were interested in whether or not a participant’s current social and economic situation influenced hunting behavior, we re-defined “hunter” as any individual who reported killing an animal in the past year, excluding one individual who reported killing a single snake on his farm. Enrollment was restricted to men because women in this area do not hunt.

### Ethics Statement

Nigeria National Parks Service and the University of Wisconsin-Madison Institutional Review Board (protocol #SE-2011-0859) approved all research activities. With the help of two Nigerian assistants, we translated all documents (site visit script, consent form, and questionnaire) into Nigerian Pidgin English. All participants provided informed oral consent. We did not obtain written consent because of low literacy rates, and because of concerns about confidentiality. We documented oral consent with the signature of the individual responsible for obtaining consent.

### Questionnaire

We designed and administered a four-part questionnaire to obtain basic demographic information, information on exposure to animals, views on the merits of hunting as a livelihood, and perceptions of zoonotic risk. Questionnaires were informed by similar studies [30,35,38], which provided the basis for establishing categories of contact modes. Local translators back-translated documents to validate the survey instrument for each village. We collected information to identify socioeconomic factors that may put individuals at risk of zoonotic infections from wild animals through hunting. These data included: age (*years*); marital status (*number of wives*); children (*number*); religion (*open*); ethnic group (*open*); education [*(0) none*, *(1) primary school*, *(2) secondary school*, *(3) beyond secondary school*]; primary occupations (*top 3; open*); father is/ was a hunter (*y/n*); house roof type [*(0) vegetation*, *(1) zinc without ceiling*, *(2) zinc with ceiling*, *(3) aluminum without ceiling*, *(4) aluminum with ceiling*], house material [*(0) mud*, *(1) mud with plaster*, *(2) cement*, *(3) cement with plaster*], domestic animals [*animal type; (0) none*, *(1) 1–5*, *(2) 6–10*, *(3) >10*]; other possessions (*generator/ television/ DVD player/ CD player/ motor bike/ cell phone*).

To assess contact frequency by species, we showed each participant published drawings of local wildlife [39], and referred to their local or English names (S1 Table). For each animal, we asked participants how often they consumed, hunted, sold, received an injury from, collected if they found dead, or kept it as a pet. These behaviors are termed “risky” throughout, as they result in direct contact between humans and wildlife species. Frequency data, unless otherwise indicated, were collected on a six-level ordinal scale (*never*, *1–5 times in their lifetime*, *1–2 times/year*, *1–2 times/ month*, *1–2 times/week or daily*). The following additional data were collected from each participant: meat preference (*bushmeat/domestic meat/ top 3 preferred wild animals*), domestic and bushmeat consumption (*frequency*), whether they: butchered bushmeat to sell (*y/n/average price*); accidently cut themselves while butchering (*y/n*); received an injury from a wild animal (y/n); used bushmeat for medicinal purposes (*y/n; animal type; description of use*); adhered to local taboos or laws against killing and/or consuming wild animals (*y/n; examples*); and used bushmeat for cultural purposes (*y/n; examples*). The following information was collected from hunters only: easiest animals to hunt (*top 3; open*), most desirable prey (*top 3; open*); hunting technique (*gun/ trap/ machete/dog*), hunting location (*forest/farm/both*), hunting time (*night/day/both*), hunting season (*occasional/wet/dry/all year*), hunting frequency, and whether they slept in the forest while on hunting excursions (*frequency*).

To characterize individual views of participants on the merits of hunting as a livelihood, we asked hunters whether they would still hunt if they had alternatives (*y/n/sometimes)*, and if they wanted their children to hunt (*y/n; why or why not*). Finally, to measure zoonotic disease awareness, perceived risk, and precautions taken to mitigate exposure, we collected data on knowledge of wildlife zoonosis (*y/n; types of diseases; source animals*); source of information (*open*); perceived threat (*y/n*); and precautionary measures taken (*y/n; explain*).

### Analyses

We used data on roofing material, housing material and household assets to create an index of household wealth. This index was based on published results of participatory and small-scale survey research comparing livelihood data for a range of households relying on non-timber forest goods in West and Central Africa [40]. Specifically, we assigned points based on roofing material (0–4), housing material (0–3), number of livestock (0–3), and non-essential household items (0–6). Thus the maximum possible score was sixteen; the higher the score, the wealthier the household.

For certain analyses we converted hunting and consumption frequencies from ordinal indices to conservative numeric estimates of minimum yearly off-take, in units of numbers of animals (never = 0, rarely = 0, yearly = 1, monthly = 12, weekly = 52 and daily = 104) for incorporation into generalized linear models. For less frequent behaviors (collect dead, injured by, kept as pet), we used the total number of animals contacted over the participant’s lifetime. We omitted cases with missing values from our analyses.

For hunters who reported hunting daily, weekly, or monthly but only during one season, we corrected hunting frequency estimates by one-half (seasonal hunters) or one-third (occasional hunters). To determine whether there were significant differences in reported contact between hunters and non-hunters, we used chi-square tests. For modes of contact where most participants engaged in the specific behavior, we compared ordinal frequencies of contact using Mann-Whitney U tests. We constructed generalized linear mixed models to examine behavioral and socioeconomic predictors of individual hunting activity and frequency of contact with wildlife. Since modes of contact are not mutually exclusive (e.g. animals can be hunted and sold or hunted and consumed), and covariance among these factors makes it difficult to separate the effect of any single behavior on overall risk, we limited our analyses of behavioral and socio-economic predictors of risk to hunting behavior alone.

To identify hunting behaviors significantly associated with high frequency of contact with all wild animals, and with specific taxa, we used mixed effects linear regression models with backwards elimination of behavioral predictor variables. We then used the same selection method in a mixed effects logistic regression model to determine which socioeconomic variables were significantly associated with being a hunter. We incorporated village as a random effect in all models. We performed analyses with *nlme* and *glmer* functions in RGui (3.0.2)[41]. We initially included all variables in the models; however, we retained only significant variables (at the alpha = .05 level) and first-order interactions among significant main effects in the final model.

## Results

### Demographic Information

Demographic information was collected from 327 individuals, representing 188 hunters and 139 non-hunters. The median age of all participants was 31.5 (range = 15–93) years. Fifty percent (n = 163/323) of individuals had the equivalent of a primary school education or lower, 32% had finished secondary school, and 18% had at least one year of higher education. Sixty-nine percent (n = 223/325) of individuals were married, and 7% had multiple wives. The average number of children was four (range = 0–26). The study populations were predominantly Christian (93%, n = 302/324), with the remainder practicing traditional religions (6%) or Islam (1%). Participants identified their tribal affiliations primarily as Efik (73%), Ejagham (14%), and a variety of other cultural groups (primarily Ibibio from neighboring Akwa Ibom state) (13%). Farming (subsistence agriculture and selling of crops) was the most common occupation (69%). Hunting (33%) and trapping (19%) were the second and third most common occupations, followed by salaried work (15%), being in school (12%), having a skilled trade (10%), selling goods (7%), driving a motorbike taxi (5%), being a village leader, being unemployed, collecting forest goods, being a member of the clergy, collecting palm wine, fishing, and livestock farming (each less than 5%).

### Contact with Wildlife

**Fig 2:**
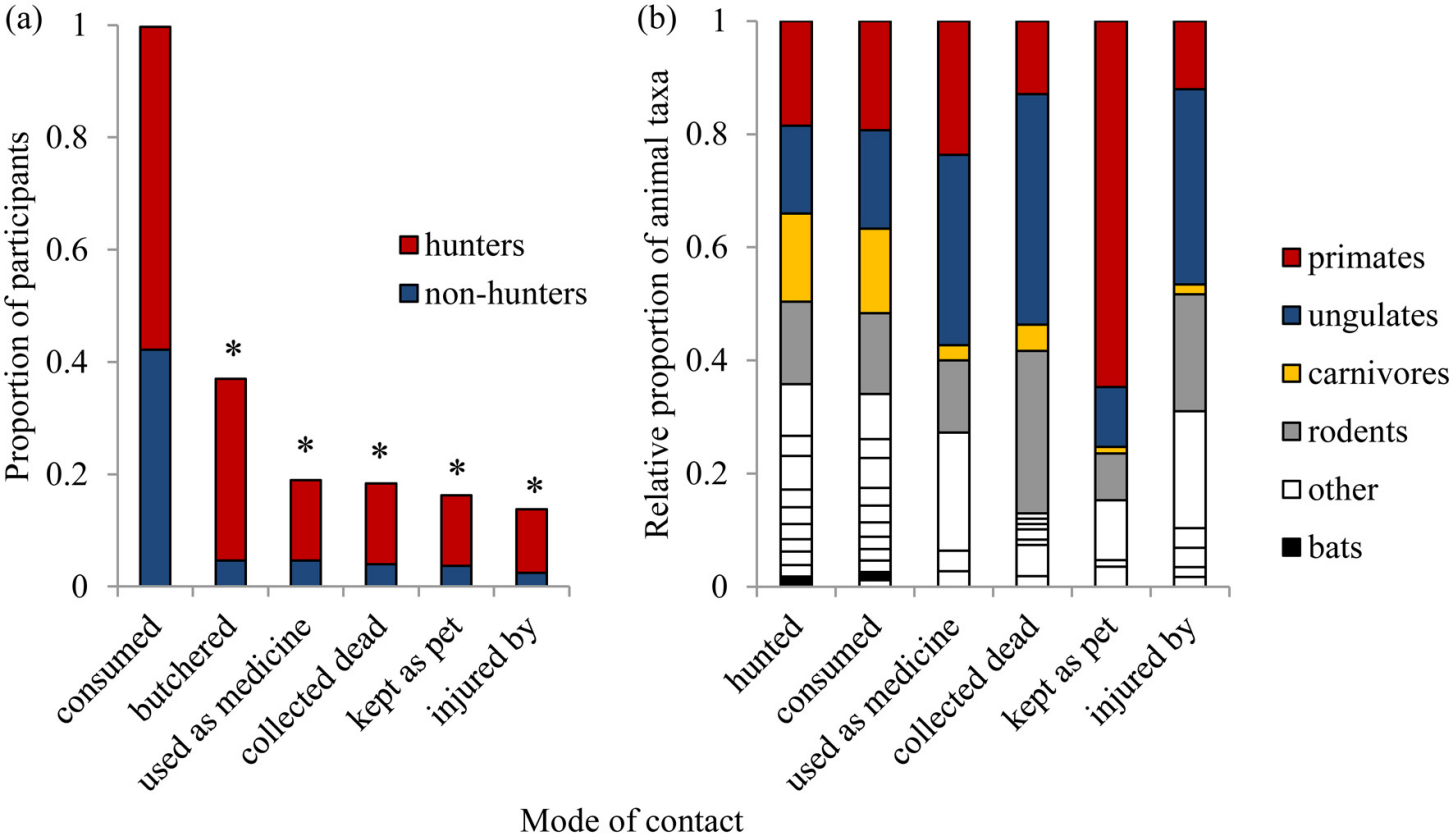
Human-wildlife contact. The proportion of participants who reported animal contact, comparing hunters (n = 188) and non-hunters (n = 137) (a), and the relative proportion of animals they reported contacted with through multiple modes (b). Asterisk indicates statistical significance at p<.05.

Over 99% of participants reported consuming bushmeat at some time in their lives. The study population, in aggregate, reported hunting and/or consuming all animals included in the survey (S2 Table). Brush-tailed porcupine (*Atherurus africanus)* was listed as the most preferred animal (45% of participants), followed by pangolin (*Manis* spp.; 16%), and monkey (*Cercocebus torquatus*, *Mandrillus leucophaeus*, and *Cercopithecus* spp.; 14%). Participants reported consuming monkeys more than once per week, and porcupine and blue duiker (*Cephalophus monticola*) slightly less than once per week. Participants also contacted wild animals through: butchering to sell (37%, n = 121), being injured (14%, n = 81), using body parts for traditional medicine (19%, n = 62), collecting carcasses found in forests and/or farms (18%, n = 60) and keeping as pets (16%, n = 53) (Fig 2a). Sixteen percent of participants reported accidently cutting themselves while butchering meat. Monkeys were reported as most frequently used for medicinal purposes (24%), followed by water chevrotain (*Hyemoschus aquaticus*; 16%) and rock python (*Python sebae*; 14%) (S3 Table). Putty-nosed guenons (*Cercopithecus nictitans*) were reported as most frequently kept as pets (23%), followed by pangolin (15%) and mona monkey (*Cercopithecus mona*) (13%). Overall, participants reported contacting primates more frequently than any other taxon (19% of “yes” responses across all species and contact modes), followed by ungulates (17%), rodents and carnivores (14% each) (Fig 2b).

Hunters were more likely than non-hunters to have reported contacting wildlife through butchering (χ^2^ = 43.67, df = 1, p<.0001), injury (χ^2^ = 46.7, df = 1, p<.0001), traditional medicine use (χ^2^ = 7.78, df = 1, p<.01), collecting carcasses (χ^2^ = 9.83, df = 1, p<.01) and keeping as pets (χ^2^ = 7.76, df = 1, p<.01) (Fig 2a). Although 99% of participants reported consuming wildlife, hunters did so more frequently (multiple times a week) than non-hunters (weekly) (U = 9287.5, p<.0001).

Seventy-five percent of participants reported consuming wildlife for cultural purposes, including festivals, holidays and special occasions. Eight percent of participants reported a taboo that prevented them from killing or consuming a certain wild animal. Taboos were typically due to family traditions (72%) or views that animals contain the spirits of ancestors (28%). Twelve percent of individuals reported that laws of the Nigerian government or local communities prevented them from killing certain animals (primates and /or endangered species) or in restricted areas.

### Hunting Behaviors

Participants reported having hunted rodents (95%), ungulates (93%), carnivores (93%), primates (87%), and bats (42%), among other prey (S2 Table). Hunters reported hunting monkeys on average more than once a week, porcupine approximately once a week, and blue duiker less than once a week over the past year. Porcupine was mentioned 47% of the time as being easiest to kill, followed by blue duiker (19%), cane rat (*Thryonomys swinderianus*) (8%), monkey (8%), and giant pouched rat (*Cricetomys emini*) (6%). Porcupine was also the most frequently mentioned (26%) as a very desirable animal, followed by red river hog (*Potamochoerus porcus*) (22%), blue duiker (17%), bay duiker (*Cephalophus dorsalis*) (8%) and monkey (7%).

Hunters used a variety of techniques, including: traps (75%), guns (71%), machetes (71%) and dogs (18%). A majority of hunters hunted only in the forest (56%), while others hunted in both the forest and on their farms (22%) or strictly on their farms (16%). Most hunted equally during the night and day (58%), but some hunted solely by day (24%) or by night (16%). The mean experience level of hunters was 11 years (range = 1–50). Seventy-five percent of hunters hunted year-round, 21% hunted in the wet season only, and 5% hunted occasionally throughout the year. On average, hunters hunted on a weekly basis, and 73% reported having slept in the forest during hunting trips.

High rates of contact with wildlife through hunting were statistically significantly associated with hunting during night and day, high hunting frequency, and hunting with a gun and dog. The frequency of primate hunting was positively associated with frequency of sleeping in the forest and time of day of hunting (night and both day and night). The frequency of hunting rodents was associated with using a gun, hunting during both the day and night, and high hunting frequency. The frequency of hunting ungulates was associated with hunting in the forest, using a machete, using a trap, and high hunting frequency. Hunting with a dog was associated with high contact with all taxa, except rodents (Table 1; S4 Table).

**Table 1:** Percentage of animals reported hunted with associated hunter behaviors.

	Primates		Rodents		Ungulates		Carnivores		All taxa	
*Behavior*	%		%		%		%		%	
Hunt often (≥ than once per week)	51.0		39.8	*	44.4	*	39.9		43.6	*
Sleep in forest often (≥ than once per week)	30.3	*	24.4		22.4		20.6		24.9	
Hunting location										
Forest	59.8		63.5		70.5	*	65.3		66.5	
Farm	17.0		13.7		10.3		13.7		11.8	
Both	23.3		22.7		19.2		21.1		21.7	
Time of day										
Day only	8.6		20.6		20		19.6		17.9	
Night only	10.8	*	13.2		10.3		13		11.8	*
Both	80.6	*	66.2	*	69.6		67.4		70.3	*
Hunts with machete	85.2		80.0		83.7	*	83.3		82	
Hunts with trap	90.5		83.7		86	*	85.5		85.2	
Hunts with gun	73.6		78.2	*	74		75.8		76.2	*
Hunts with dog	35.7	*	33.0		40.6	*	34.7	*	36.3	*

* p-value with significance at ≤ .05 based on multiple linear mixed effects regression models predicting reported hunting frequencies (S4 Table).

### Reasons for Hunting

Overall, participants reported a strong preference (84%) for bushmeat over domestic meat. Participants hunted to both sell and eat meat (73%), although some hunted exclusively for household consumption (22%) or exclusively to sell (5%). The top five preferred animals and average market price (in USD per animal, converted from 2012 exchange rates from Nigerian Naira to US Dollars), were: 1) porcupine (42%; $16); 2) pangolin (15%; $8); 3) monkey (11%; $15); 4) red-river hog (8%; $106); and 5) blue duiker (4%; $17). Seventy-five percent (n = 111/156) of hunters had fathers who were also hunters.

Eighty-four percent (n = 145/173) of participants reported that they would choose not to hunt if they had an alternative source of income. Ninety-seven percent of participants reported not wanting their children to hunt. The most common reason that people gave for not wanting their children to hunt was that hunting was too difficult (49%; n = 159/324). Thirty-eight percent of respondents used the word “suffer” or “stress” to explain why they did not want their children to hunt. Other common reasons were that hunting was too dangerous (15%), was not a real job (10%) or that it was no longer as profitable due to declining wildlife numbers (7%). Education and age were negatively associated with becoming a hunter, whereas household size, having a father who hunts, and being of the resident cultural group were significantly associated with becoming a hunter (Table 2).

**Table 2:** Factors associated with hunting.

Factor*	OR (95% CI)	*p-value*
Age (years)	0.96 (0.93–0.99)	<.01
Education		
*none (reference)*	—	—
*primary school*	0.61 (0.20–1.82)	ns
*secondary school*	0.30 (0.10–0.93)	<.05
*post-secondary school*	0.19 (0.06–0.62)	<.01
Household size (# of individuals)	1.20 (1.08–1.32)	<.001
Father hunts (yes versus no)	2.86 (1.57–5.26)	<.001
Resident cultural group (yes versus no)	4.14 (1.50–11.50)	<.01

* Village was incorporated as a random effect into a logistic regression model. There was no effect of wealth on whether or not an individual hunted.

### Zoonotic Disease Awareness

**Fig 3:**
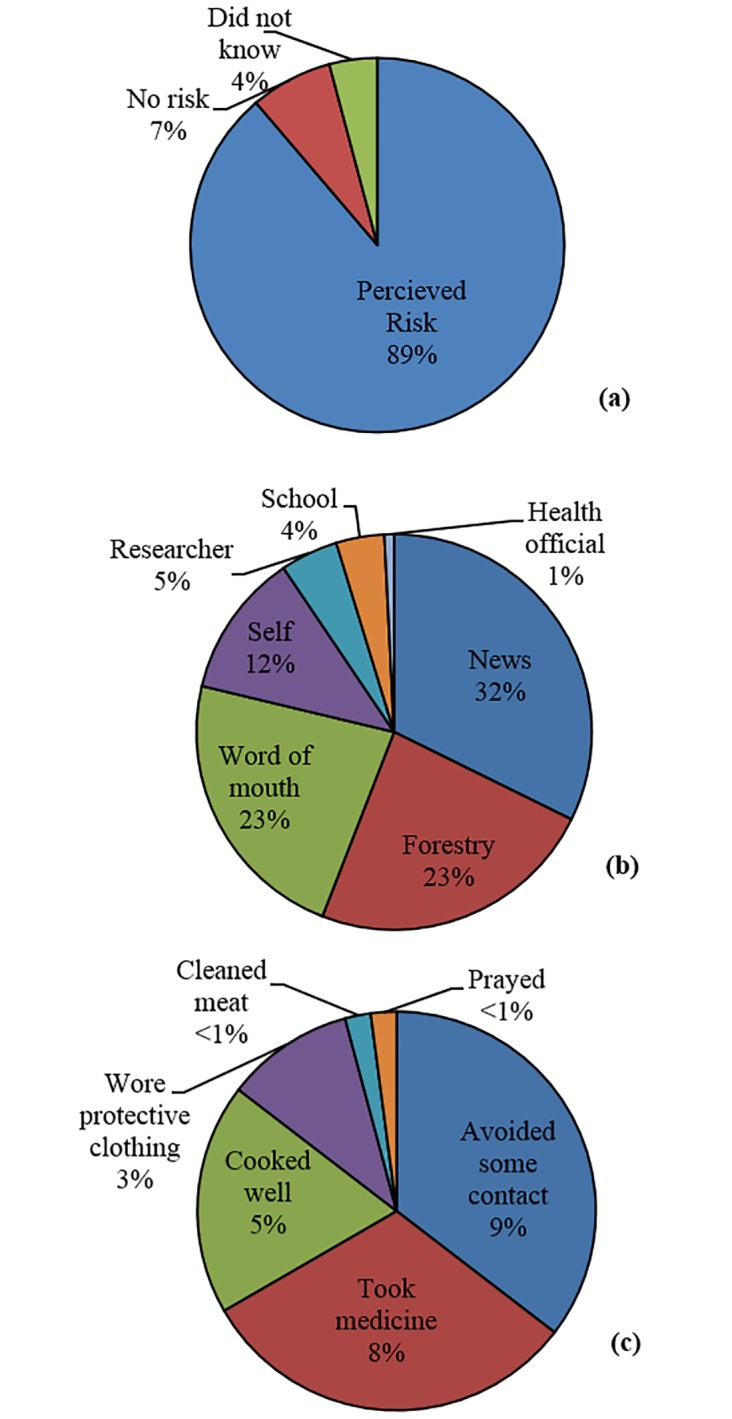
Perceptions of zoonotic disease risk. Level of perceived risk (a), sources of information (b), and protective behaviors (c), of the 55% of participants who reported awareness of wildlife zoonoses.

Fifty-five percent of participants reported awareness of wildlife zoonoses, with information spread primarily through broadcast news outlets, forestry/ conservation workers, or word of mouth. Of the individuals reporting awareness of zoonoses, 89% said that they perceived an actual risk and 26% reported taking measures to protect themselves from infection (Fig 3). Participants described 21 diseases that they believed came from wild animals: HIV (55%), cough (11%), malaria (5%), poison (5%), tumbu flies (*Cordylobia anthropophaga*, a parasitic fly; 4%), flu, gonorrhea, body pain, sleeping sickness (2% each), typhoid, fever, rash, cholera, scabies, worms, SARS, rickets, boil, typhus, syphilis, rabies (each 1%). Wild animals believed to be responsible to zoonotic infections included: monkey (55%), python (12%), red-river hog (10%), chimpanzee (7%), leopard (5%) and duiker (4%).

## Discussion

We found that younger age, lower education level, larger household size, having a father who hunts, and being of the resident cultural group were all significantly associated with becoming a hunter. Hunters had more frequent contact with wildlife through both hunting and non-hunting behaviors, likely experiencing higher exposure risk to zoonosis than non-hunters. Specific hunting behaviors, namely high hunting frequency, hunting during both day and night, hunting specifically at night, and hunting with a gun and with a dog were all associated with high rates of contact with wildlife. Other behaviors were associated with higher rates of contact with specific taxa, namely: sleeping in the forest (primates), and using a machete and trap (ungulates). Carnivore and rodent hunting frequency was not uniquely associated with any specific hunting behaviors.

Our results shed new light on the social-cultural contexts of wildlife contact in this region and have implications for conservation and public health. We found a negative association between education level and hunting, and no effect of wealth. These results differ from those of Le Breton and colleagues (2006) who found that hunting in Cameroon was more common among poorer households (as measured by roof type) with no effect of education level. Similarly, in Tanzania, participation in illegal hunting decreased with increasing wealth, as measured by ownership of sheep and goats [28]. Negative results in our study may reflect low variation in economic status, in that all participants were almost uniformly materially disadvantaged. This conclusion is supported by our observation that larger family sizes appeared to generate a greater need for income, which may be most accessible through hunting, particularly for individuals from families with experienced hunters. Individuals with higher education levels, a factor associated with lower probability of hunting in our study, do not necessarily have higher income, but may engage in activities that generate extra income or in other commitments that keep them out of the forest.

Our data also show that resident cultural groups were more likely to hunt than other cultural groups, which tend to be migrants from nearby states. Our study sites varied in numbers of migrants from neighboring states, but when present, they commonly resided in the periphery of villages as farmers and were not permitted to hunt by order of village chiefs. Our results contrast those of other studies that found that migrants hunted a majority of bushmeat (Congo), had higher rates of primate contact (Uganda), and were more likely to be involved in butchering (Cameroon) than resident groups [12,30,42]. Our results may reflect cultural differences among migrant populations, or a unique local response from resident cultural groups who fear loss of livelihoods to migrant populations.

Although hunting is illegal and considered an undesirable livelihood, strong incentives to hunt still persist. Indeed, we struggled to identify men who had never hunted or trapped wildlife. Nevertheless, almost all participants claimed that if given an alternative, they would choose not to hunt. Virtually all said they did not want their children to become hunters because it is too difficult, dangerous, stressful, not a legitimate occupation, and is no longer profitable. This contrasts directly with historical accounts of hunting in this region, in which hunters were described as being “economically independent” and “far too important a person” to employ [43; pg.152]. We suggest that declines in wildlife numbers coupled with increasing distances between wildlife habitat and villages have decreased incentives to hunt. With lower returns per hunt, those who are able turn to alternatives. Many who continue to hunt do so out of necessity, and in turn, hunting is viewed as a low-merit livelihood, even among hunters themselves. While preference for bushmeat will inevitably drive the trade to a certain degree, our data suggest that provision of alternative livelihoods would reduce hunting behavior by restricting hunting frequency and providing supplemental income. However, individuals in need of extra income would remain free to hunt at night and set traps, which were predictors of primate and ungulate hunting frequency, respectively.

Nearly all participants reported consuming bushmeat, and there was a strong preference for bushmeat over domestic meat. A majority of participants had strong cultural ties to the consumption of bushmeat, and very few recognized laws protecting wildlife. A majority of hunters reported selling bushmeat, indicating that demand from rural and urban markets continue to provide incentives for bushmeat hunters, who often lack alternative ways to generate income. These incentives may be modified by hunting taboos. For example, in neighboring states in Nigeria, certain guenons are held as deities and are protected within particular villages [44]. In this region however, such taboos were uncommon. As a result, hunting in and near protected areas remains common in Nigeria [13,45].

Although previous studies reported frequencies of carcasses in bushmeat markets in this region [13], we are aware of none examining hunting preferences and cultural uses that may be driving human-wildlife contact at the local level. Significantly, we found that participants reported having had contact with primates more than with any other wildlife taxon. This remains true whether keeping wildlife as pets is included in our analyses, since it was reported far less frequently than hunting. However, we note that keeping animals as pets presents a different kind of risk, in which people come into frequent contact with animals over a prolonged period. This behavior, unlike others, leads to opportunities for repeated injury and exposure to animals that may be persistently stressed.

Eighty-seven percent of individuals reported consuming primates, and monkeys were listed among the most desirable animals to eat and were most frequently mentioned as useful for medicinal purposes or kept as a pet. These data parallel high primate consumption rates [30], and preferences for primates [35] documented for other regions. Porcupine and blue duiker were consumed by over 90% of individuals, with porcupine most frequently mentioned as a preferred meat. Ebola epidemics have been previously associated with handling duiker carcasses [46], and though we are unaware of zoonotic viruses transmitted directly through contact with porcupines, rodents in general host more than 60 known zoonotic viruses [17]. Bats, along with other small prey, were anecdotally referred to as “children’s meat”, in that they are small and thus given to children to play with and eat, thereby potentially putting children at greater risk. The link between bushmeat hunting and zoonotic disease risk through such pathways has been discussed extensively [17,18,30]; our data expand these risks to a new region and a new cultural setting.

Of the 55% of participants who reported awareness of zoonotic diseases from wildlife, a majority reported believing that there was an actual risk associated with contact. Awareness of wildlife zoonoses was considerably higher than reported in hunting communities in Sierra Leone ([35]; 55% *versus* 24%), but overall perceived risk was lower than in Cameroon ([30]; 46% *versus* 74%). Differences across study sites may be due to educational campaigns in the respective areas. We are unaware of public health outreach campaigns related to wildlife and disease in this region. However, given the proximity to the national park, participants may have previously received information of risks associated with hunting and consumption of wildlife species of conservation concern, particularly primates. In our study, information about risk came primarily through broadcast news outlets, forestry/conservation personnel, and word of mouth. Only one individual reported a public health official as a source of information about zoonotic diseases, despite the fact that such individuals are in strong positions to enhance knowledge of risks associated with bushmeat, especially near protect areas where wildlife contact rates are high.

Fifty-five percent of participants who reported awareness of wildlife zoonoses gave monkeys and HIV as an example. However, many other examples were of unconfirmed hosts or non-zoonotic pathogens. Despite such knowledge, very few individuals reported protecting themselves from infection. Avoidance was the most frequently cited protective measure, including avoidance of eating bushmeat, touching blood, sexual contact, or eating fruit from trees where monkeys had been feeding. Of those who protected themselves, 31% reported taking traditional and/or commercial medicine as a treatment or prophylaxis. Many potential zoonoses are viral and therefore locally available treatments such as saline injections, antibiotics and acetaminophen would be ineffective. Additionally, the effectiveness of traditional treatments such as consumption of wild herbs or bitter cola (*Garcinia afzelii*) against zoonotic pathogens is as yet unproven. Only five percent of participants reported using safe meat handling practices, such as cleaning or cooking meat well prior to consumption, as a protective measure. We recorded differences in consumption patterns among locations (e.g. consumption of partially smoked innards at the hunting sheds *versus* well-smoked meat sold in markets), suggesting that risk of contact and zoonoses varies across space and time. Participants also reported wearing clothes and/or boots for protection, for example when hunters carry carcasses over long distances (wearing clothing) or restrain animals with their feet (wearing boots). One participant reported wearing protective gloves while butchering. The efficacy of these measures for protecting against exposure to infectious material is unknown, but is likely to be higher than using no protection at all. Education programs implemented through conservation programs and/or news outlets should therefore include information on avoidance strategies, with specific attention to dispelling misconceptions about routes of transmission and promoting effective and accessible strategies for mitigating exposure.

Our findings highlight the value of understanding socio-cultural drivers of bushmeat hunting for reducing contact with wildlife in high-risk groups. Hunting wildlife for meat is widespread in West and Central Africa, and effective public health solutions are unlikely to emerge from conservation and regulatory agencies alone. Our data suggest that effective solutions will include implementation of alternative livelihood programs specifically targeting hunters and aimed at providing alternative protein sources that would satisfy local taste preferences (e.g. raising desirable species in captivity [47,48]). Conservation rules that limit hunting, or prohibit hunting with dogs, and are implemented with the help of local chiefs may be most effective in reducing hunting pressure, such as in the case of effectively restricting hunting privileges to resident groups. However, given the cultural and economic contexts of the bushmeat trade, a complete shift to alternative protein sources may be impractical at present.

Novel self-protective strategies should be developed through consultation with individuals who currently protect themselves, make use of locally available goods, and be tested locally for cultural acceptability. Our data suggest that conservation and public health initiatives that tap into existing outlets for transmitting information, such as word of mouth and radio broadcasting, are likely to be most effective in reaching and influencing people in high risk areas. Although our study focuses on drivers of hunting, a behavior practiced only by men in this region, women are at risk from butchering and trading the animals brought back by hunters [35,49], and should also be targeted during educational programs and interventions.

Results from Nigeria demonstrate that hunters in this setting frequently contact a diversity of prey in “risky” ways, and that the decision to become a hunter is rooted in family tradition, modified by economic necessity. Improved education, reduced family sizes, and provision of alternative livelihoods may result in reduced contact with wildlife and lower zoonotic disease risk in rural hunting communities in Nigeria and similar locations. We acknowledge that such solutions require the mobilization of significant resources toward development and conservation jointly. We also advocate targeting neglected transmission pathways, such as distinct cultural uses of wildlife that motivate off-take and provide novel routes for pathogen exchange. These potential routes of transmission have received less attention than those associated with hunting of bushmeat for consumption, but they may in aggregate confer equal or greater risk.

## Supporting Information

S1 Table Classifications of contacted animals. (DOCX)

S2 Table Summary of wild animals hunted and consumed. (DOCX)

S3 Table Summary of wild animals used as traditional medicine. (DOCX)

S4 Table Behavioral predictors of contact with wildlife taxa. (DOCX)

S1 Checklist STROBE checklist. (DOC)

## References

[1] Wilkie DS, Carpenter JF. Bushmeat hunting in the Congo Basin: an assessment of impacts and options for mitigation. Biodivers Conserv. 1999;8: 927–955.

[2] Fa JE, Peres CA, Meeuwig J. Bushmeat exploitation in tropical forests: an intercontinental comparison. Conserv Biol. 2002;16: 232–237. 10.1046/j.1523-1739.2002.00275.x 35701970

[3] Karesh WB, Noble E. The Bushmeat Trade: Increased Opportunities for Transmission of Zoonotic Disease. Mt Sinai J Med J Transl Pers Med. 2009;76: 429–434. 10.1002/msj.20139 19787649

[4] Bennett E, Robinson J, editors. Hunting for Sustainability in Tropical Forests. New York: Columbia University Press; 1999 10.1111/j.1523-1739.2009.01224.x

[5] Milner-Gulland EJ, Bennett EL. Wild meat: the bigger picture. Trends Ecol Evol. 2003;18: 351–357. 10.1016/S0169-5347(03)00123-X

[6] Brown D, Davies G, editors. Bushmeat and livelihoods: wildlife management and poverty reduction. Oxford: Blackwell Publishing Ltd; 2007.

[7] Wolfe ND, Daszak P, Kilpatrick AM, Burke DS. Bushmeat hunting, deforestation, and prediction of zoonotic disease. Emerg Infect Dis. 2005;11: 1822–1827. 16485465 10.3201/eid1112.040789 PMC3367616

[8] Hart JA. From subsistence to market: a case study of the Mbuti net hunters. Hum Ecol. 1978;6: 325–353. 10.1007/BF00889029

[9] Hart TB, Hart JA. The ecological basis of hunter-gatherer subsistence in African rain forests: the Mbuti of Eastern Zaire. Hum Ecol. 1986;14: 29–55.

[10] Wilkie DS, Sidle JG, Boundzanga GC. Mechanized logging, market hunting, and a bank loan in Congo. Conserv Biol. 1992;6: 570–580. 10.1046/j.1523-1739.1992.06040570.x

[11] Bowen-Jones E, Pendry S. The threat to primates and other mammals from the bushmeat trade in Africa, and how this threat could be diminished. Oryx. 1999;33: 233–246. 10.1046/j.1365-3008.1999.00066.x

[12] Poulsen JR, Clark CJ, Mavah G, Elkan PW. Bushmeat supply and consumption in a tropical logging concession in northern Congo. Conserv Biol. 2009;23: 1597–1608. 10.1111/j.1523-1739.2009.01251.x 19459888

[13] Fa JE, Seymour S, Dupain J, Amin R, Albrechtsen L, Macdonald D. Getting to grips with the magnitude of exploitation: bushmeat in the Cross—Sanaga rivers region, Nigeria and Cameroon. Biol Conserv. 2006;129: 497–510. 10.1016/j.biocon.2005.11.031

[14] Chaber A-L, Allebone-Webb S, Lignereux Y, Cunningham AA, Marcus Rowcliffe J. The scale of illegal meat importation from Africa to Europe via Paris. Conserv Lett. 2010;3: 317–321. 10.1111/j.1755-263X.2010.00121.x

[15] Toledo LF, Asmüssen MV, Rodríguez JP. Track illegal trade in wildlife. Nature. 2012;483: 36 10.1038/483036e 22382968

[16] LeBreton M, Pike BL, Saylors KE, Diffo JLD, Fair JN, Rimoin AW, et al Bushmeat and infectious disease emergence In: Aguirre AA, Ostfeld R, Daszak P, editors. New directions in conservation medicine: applied cases of ecological health. Oxford; New York: Oxford University Press; 2012.

[17] Luis AD, Hayman DTS, O’Shea TJ, Cryan PM, Gilbert AT, Pulliam JRC, et al A comparison of bats and rodents as reservoirs of zoonotic viruses: are bats special? Proc R Soc Lond B Biol Sci. 2013;280: 20122753 10.1098/rspb.2012.2753 PMC3574368 23378666

[18] Meerburg BG, Singleton GR, Kijlstra A. Rodent-borne diseases and their risks for public health. Crit Rev Microbiol. 2009;35: 221–270. 10.1080/10408410902989837 19548807

[19] Pedersen AB, Davies TJ. Cross-species pathogen transmission and disease emergence in primates. EcoHealth. 2009;6: 496–508. 10.1007/s10393-010-0284-3 20232229 PMC7087625

[20] Davies TJ, Pedersen AB. Phylogeny and geography predict pathogen community similarity in wild primates and humans. Proc R Soc B Biol Sci. 2008;275: 1695–1701. 10.1098/rspb.2008.0284 18445561 PMC2602822

[21] Sharp PM, Hahn BH. Origins of HIV and the AIDS pandemic. Cold Spring Harb Perspect Med. 2011;1: 1–22. 10.1101/cshperspect.a006841 PMC3234451 22229120

[22] Peeters M, D’Arc M, Delaporte E. Origin and diversity of human retroviruses. AIDS Rev. 2014;16: 23–34. 24584106 PMC4289907

[23] Huchon D, Madsen O, Sibbald MJJB, Ament K, Stanhope MJ, Catzeflis F, et al Rodent Phylogeny and a Timescale for the Evolution of Glires: Evidence from an Extensive Taxon Sampling Using Three Nuclear Genes. Mol Biol Evol. 2002;19: 1053–1065. 12082125 10.1093/oxfordjournals.molbev.a004164

[24] Wilson DE, Reeder DM. Mammal species of the world. Smithsonian Institution Press; 1993.

[25] Plowright RK, Eby P, Hudson PJ, Smith IL, Westcott D, Bryden WL, et al Ecological dynamics of emerging bat virus spillover. Proc R Soc B Biol Sci. 2015;282: 20142124 10.1098/rspb.2014.2124 25392474 PMC4262174

[26] Chomel BB, Belotto A, Meslin F-X. Wildlife, exotic pets, and emerging zoonoses. Emerg Infect Dis. 2007;13: 6 17370509 10.3201/eid1301.060480 PMC2725831

[27] Dei GJS. Hunting and gathering in a Ghanaian rain forest community. Ecol Food Nutr. 1989;22: 225–243. 10.1080/03670244.1989.9991071

[28] Loibooki M, Hofer H, Campbell KLI, East ML. Bushmeat hunting by communities adjacent to the Serengeti National Park, Tanzania: the importance of livestock ownership and alternative sources of protein and income. Environ Conserv. 2002;29: 391–398. 10.1017/S0376892902000279

[29] Brown D, Williams A. The case for bushmeat as a component of development policy: issues and challenges. Int For Rev. 2003;5: 148–155.

[30] LeBreton M, Prosser AT, Tamoufe U, Sateren W, Mpoudi-Ngole E, Diffo JLD, et al Patterns of bushmeat hunting and perceptions of disease risk among central African communities. Anim Conserv. 2006;9: 357–363. 10.1111/j.1469-1795.2006.00030.x

[31] Brashares JS, Golden CD, Weinbaum KZ, Barrett CB, Okello GV. Economic and geographic drivers of wildlife consumption in rural Africa. Proc Natl Acad Sci. 2011;108: 13931–13936. 10.1073/pnas.1011526108 21873180 PMC3161600

[32] Wickramasinghe A, Pérez MR, Blockhus JM. Nontimber forest product gathering in Ritigala Forest (Sri Lanka): household strategies and community differentiation. Hum Ecol. 1996;24: 493–519.

[33] De Merode E, Homewood K, Cowlishaw G. The value of bushmeat and other wild foods to rural households living in extreme poverty in Democratic Republic of Congo. Biol Conserv. 2004;118: 573–581. 10.1016/j.biocon.2003.10.005

[34] Godoy R, Undurraga EA, Wilkie D, Reyes-García V, Huanca T, Leonard WR, et al The effect of wealth and real income on wildlife consumption among native Amazonians in Bolivia: estimates of annual trends with longitudinal household data (2002–2006): Wildlife consumption, wealth and income. Anim Conserv. 2010;13: 265–274. 10.1111/j.1469-1795.2009.00330.x 21217956

[35] Subramanian M. Zoonotic disease risk and the bushmeat trade: assessing awareness among hunters and traders in Sierra Leone. EcoHealth. 2012;9: 471–482. 10.1007/s10393-012-0807-1 23408099

[36] Myers N, Mittermeier RA, Mittermeier CG, Da Fonseca GA, Kent J. Biodiversity hotspots for conservation priorities. Nature. 2000;403: 853–858. 10706275 10.1038/35002501

[37] Jones KE, Patel NG, Levy MA, Storeygard A, Balk D, Gittleman JL, et al Global trends in emerging infectious diseases. Nature. 2008;451: 990–993. 10.1038/nature06536 18288193 PMC5960580

[38] Wolfe ND, Switzer WM, Carr JK, Bhullar VB, Shanmugam V, Tamoufe U, et al Naturally acquired simian retrovirus infections in central African hunters. The Lancet. 2004;363: 932–937. 15043960 10.1016/S0140-6736(04)15787-5

[39] Kingdon J. The Kingdon pocket guide to African mammals. Princeton: Princeton University Press; 2005.

[40] Malleson R, Asaha S, Sunderland T, Burnham P, Egot M, Obeng-Okrah K, et al A methodology for assessing rural livelihood strategies in West/Central Africa: lessons from the field. Ecol Environ Anthropol Univ Ga. 2008; 1–12.

[41] R Core Team. R: A Language and Environment for Statistical Computing [Internet]. Vienna, Austria: R Foundation for Statistical Computing; 2014 Available: http://www.R-project.org

[42] Paige SB, Frost SDW, Gibson MA, Jones JH, Shankar A, Switzer WM, et al Beyond bushmeat: animal contact, injury, and zoonotic disease risk in Western Uganda. EcoHealth. 2014; 10.1007/s10393-014-0942-y PMC4240769 24845574

[43] Sanderson IT. Animal Treasure. First edition Viking Adult; 1937.

[44] Oates J. A survey of primates and other forest wildlife in Anambra, Imo and Rivers States, Nigeria. Report to National Geographic Society (USA), Nigerian Conservation Foundation, Nigerian Federal Department of Forestry, and Governments of Anambra, Imo and Rivers States.; 1989.

[45] Fa JE, Farfán MA, Marquez AL, Duarte J, Nackoney J, Hall A, et al Mapping hotspots of threatened species traded in bushmeat markets in the Cross—Sanaga rivers region. Conserv Biol. 2014;28: 224–233. 10.1111/cobi.12151 24024960

[46] Leroy EM, Rouquet P, Formenty P, Souquière S, Kilbourne A, Froment J-M, et al Multiple Ebola Virus Transmission Events and Rapid Decline of Central African Wildlife. Science. 2004;303: 387–390. 10.1126/science.1092528 14726594

[47] Jori F, Mensah GA, Adjanohoun E. Grasscutter production: an example of rational exploitation of wildlife. Biodivers Conserv. 1995;4: 257–265. 10.1007/BF00055972

[48] Jori F, Lopez-béjar M, Houben P. The biology and use of the African brush-tailed porcupine (Atherurus africanus, Gray, 1842)as a food animal. A review. Biodivers Conserv. 1998;7: 1417–1426. 10.1023/A:1008853113835

[49] Kamins AO, Rowcliffe JM, Ntiamoa-Baidu Y, Cunningham AA, Wood JLN, Restif O. Characteristics and Risk Perceptions of Ghanaians Potentially Exposed to Bat-Borne Zoonoses through Bushmeat. EcoHealth. 2014; 1–17. 10.1007/s10393-014-0977-0 PMC4416116 25266774

