# Supplementary material for: Drivers of Bushmeat Hunting and Perceptions of Zoonoses in Nigerian Hunting Communities
Source: PLoS Negl Trop Dis. 2015 May 22;9(5):e0003792. doi: 10.1371/journal.pntd.0003792 (PMC4441483; doi:10.1371/journal.pntd.0003792)
Supplement: S1 Table — (DOCX) [file pntd.0003792.s001.docx]

Table S1. Classifications of contacted animals

| English name | Scientific Name | Family | Order | Class | Local English names* | Efik dialects |  | Ejagham dialect |
| --- | --- | --- | --- | --- | --- | --- | --- | --- |
| Fruit bat | several genera | Pteropodidae | Chiroptera | Mammalia | Bat | Iyumamonc | Igegemi | Egangang |
| Squirrel | several genera | Sciuridae | Rodentia | Mammalia | Squirrel | Epang | Kwakuru | Ikomey |
| Flying squirrel | *Anomalurus* sp. | Anomaluridae | Rodentia | Mammalia | Flying rabbit | Erret | Erre | Ebaghi |
| Giant-pouched Rat | *Cricetomys emini* | Nesomyidae | Rodentia | Mammalia | Rabbit | Eboi | Ewe | Nku |
| Marsh cane-rat | *Thryonomys swinderianus* | Thryonomyidae | Rodentia | Mammalia | Cutting Grass | Ephip | Ebeck | Kobiya |
| Tree hyrax | *Dendrohyrax* *dorsalis* | Procaviidae | Hyracoidea | Mammalia | -- | Bjururum | Ekorurum | Ekpim |
| Cusimanse | *Crossarchus obscurus* | Herpestidae | Carnivora | Mammalia | -- | Boyok | Bidam | Ifet |
| Mongoose | *Herpestes sanguinea* | Herpestidae | Carnivora | Mammalia | Fox/ Bush dog | Epetam | Gowut | Ebi |
| Brush-tailed porcupine | *Atherurus africanus* | Hystricidae | Rodentia | Mammalia | Chucuchucu | Iyup | Ikup | Nyop |
| Tree pangoline | *Phataginus tricuspus* | Manidae | Pholidota | Mammalia | Pangoline/ Catta beef | Iyan | Gegang | Ika |
| Giant otter shrew | *Potamogale velox* | Tenerecidae | Afrosoricida | Mammalia | Water rabbit | Betek | Ebochabugai | Esimsor |
| African clawless otter | *Aonyx capensis* | Mustelidae | Carnivora | Mammalia | -- | Ebuboia | -- | Ikiyork |
| Common genet | *Genetta gentta* | Viverridae | Carnivora | Mammalia | Bushbaby/ Bush cat | Akokanta | Birun | Nsim |
| Palm civet | *Nandinia bibotata* | Nandiniidae | Carnivora | Mammalia | Stone beef | Udi | Inie | Mbai |
| African civet | *Civettictis civetta* | Viverridae | Carnivora | Mammalia | Bush Dog/ Hyena | Sup | Chup | Ejor |
| Golden cat | *Felis aurata* | Felidae | Carnivora | Mammalia | Lion | Ekbaiba | -- | Ekparim mgbe |
| Leopard | *Panthera pardus* | Felidae | Carnivora | Mammalia | Tiger | Ekpe | Gewaiwai | Mgbe |
| Chimpanzee | *Pan troglodytes* | Hominidae | Primates | Mammalia | Chimpanzee | Idubatam | Inumadam | Nyork |
| Drill | *Mandrillus leucophaeus* | Cercopithecidae | Primates | Mammalia | Drill | Iyum | Iyum | Nsum |
| Red-capped mangabey | *Cercocebus torquatus* | Cercopithecidae | Primates | Mammalia | Red Head | Ekpo | Iku | Mbi |
| Red colobus | *Procolobus pennatii preussi* | Cercopithecidae | Primates | Mammalia | -- | Udim | Iku | Ekabok |
| Red-eared Monkey | *Cercopithecus erythrotis* | Cercopithecidae | Primates | Mammalia | Monkey- red tail | Iona | Iku | Mbi Mbuk |
| Mona monkey | *Cercopithecus mona* | Cercopithecidae | Primates | Mammalia | Monkey | Epem | Iku | Mbarambuk |
| Putty-nosed Monkey | *Cercopithecus nictitans* | Cercopithecidae | Primates | Mammalia | Monkey- white nose | Upena | Iku | Numyak/ Nyakambuk (male/female) |
| Bushbaby | *Galago spp.* | Galagidae | Primates | Mammalia | -- | Bikboon | Gecacari | Ebop |
| Potto/ calabar Angwantibo | *Perodicticus potto/ Arctocebus calabarensis* | Lorisidae | Primates | Mammalia | Fox | Dechai | Gecat Kagon | Efe |
| Blue duiker | *Cephalophus monticola* | Bovidae | Artiodactyla | Mammalia | Frutumbo | Bituna | Biduna | Iseh |
| Red river hog | *Potamochoerus porcus* | Suidae | Artiodactyla | Mammalia | Bush Pig | Iyrre | Genibagai | Mgumi |
| Bay duiker | *Cephalophus dorsalis* | Bovidae | Artiodactyla | Mammalia | Red Deer | Ebin | Inumadam | Nsun |
| Yellow-backed duiker | *Cephalophus sivicultor* | Bovidae | Artiodactyla | Mammalia | Bush Cow | Ajima | Gemem | Ngugu |
| Water chevrotain | *Hyemoschus aquaticus* | Tragulidae | Artiodactyla | Mammalia | Water Beef | Bejuy | Eget | Iku |
| Sitatunga | *Tragelaphus spekei* | Bovidae | Artiodactyla | Mammalia | Antelope | Idup | Ochup | Ngongum |
| African buffalo | *Syncerus caffer* | Bovidae | Artiodactyla | Mammalia | Buffalo | Ebongai | Etua | Mfumg |
| African forest elephant | *Loxodonta africana cyclotis* | Elephantidae | Proboscidea | Mammalia | Elephant | Idi | Ini | Njok |
| Crocodile | *Osteolaemus tetraspis* | Crocodylidae | Crocodilia | Reptilia | Crocodile | Etararam | Gegum | Nyip |
| Tortoise | *Kinixys sp.* | Testudinidae | Testudines | Reptilia | Tortoise | Bejin | Gegen | Nkui |
| Monitor lizard | *Varanus sp.* | Varanidae | Squamata | Reptilia | Iguna | Uti | Gowen | Ebak |
| Black cobra | *Naja melanoleuca* | Elapidae | Squamata | Reptilia | cobra | Ugwema | Ugema | Nyor Nyak |
| Rock python | *Python sebae* | Boidae | Squamata | Reptilia | Snake | Etan | Edang | Nkum |
| Great blue turaco | *Corythaeola cristata* | Musophagidae | Musophag-iformes | Aves | -- | Okonoc | Kundok | Nkurak |
| Hornbill | several genera | Bucerotidae | Bucerotiformes | Aves | -- | Etogi | Gedobi | Mgon |
| Black guinea fowl | Agelastes *niger* | Numididae | Galliformes | Aves | -- | Etamphana odone | Genon bana | Eviechi |
| Guinea fowl | *Guttera plumifera* | Numididae | Galliformes | Aves | -- | Otamma | Gedami | Enyeng |

* Blanks indicate that there was no name for the animal in English, Efik or Ejagham
