# Supplementary material for: Drivers of Bushmeat Hunting and Perceptions of Zoonoses in Nigerian Hunting Communities
Source: PLoS Negl Trop Dis. 2015 May 22;9(5):e0003792. doi: 10.1371/journal.pntd.0003792 (PMC4441483; doi:10.1371/journal.pntd.0003792)
Supplement: S2 Table — (DOCX) [file pntd.0003792.s002.docx]

| Table S2. Summary of wild animals hunted and consumed | | | |
| --- | --- | --- | --- |
| Order | Species | Hunted^a^ | Consumed^b^ |
| Squamata |  | **0.97** | **0.86** |
|  | Monitor lizard (*Varanus sp.*) | 0.83 | 0.76 |
|  | Rock python (*Python sebae*) | 0.74 | 0.71 |
|  | Black cobra (*Naja melanoleuca*) | 0.65 | 0.50 |
| Rodentia |  | **0.95** | **0.99** |
|  | Brush-tailed porcupine (*Atherurus africanus*) | 0.93 | 0.98 |
|  | Marsh cane-rat (*Thryonomys swinderianus*) | 0.84 | 0.86 |
|  | Giant-pouched rat (*Cricetomys emini*) | 0.84 | 0.81 |
|  | Squirrel (Family: Sciuridae) | 0.64 | 0.58 |
|  | Flying squirrel (*Anomalurus* sp.) | 0.30 | 0.28 |
| Artiodactyla |  | **0.93** | **0.96** |
|  | Blue duiker (*Cephalophus monticola*) | 0.92 | 0.94 |
|  | Bay duiker (*Cephalophus dorsalis*) | 0.78 | 0.83 |
|  | Red-river hog (*Potamochoerus porcus*) | 0.67 | 0.83 |
|  | Sitatunga (*Tragelaphus spekei*) | 0.57 | 0.63 |
|  | Water chevrotain (*Hyemoschus aquaticus*) | 0.55 | 0.55 |
|  | Yellow-backed duiker (*Cephalophus sivicultor*) | 0.23 | 0.36 |
|  | African buffalo (*Syncerus caffer*) | 0.04 | 0.14 |
| Carnivora |  | **0.93** | **0.91** |
|  | Cusimanse (*Crossarchus obscurus*) | 0.81 | 0.78 |
|  | Common genet (*Genetta gentta*) | 0.80 | 0.71 |
|  | Palm civet (*Nandinia bibotata*) | 0.77 | 0.73 |
|  | Mongoose (*Herpestes sanguinea*) | 0.69 | 0.61 |
|  | African civet (*Civettictis civetta*) | 0.44 | 0.50 |
|  | African clawless otter (*Aonyx capensis*) | 0.26 | 0.25 |
|  | Golden cat (*Felis aurata*) | 0.03 | 0.03 |
|  | Leopard (*Panthera pardus*) | 0.01 | 0.06 |
| Primates |  | **0.87** | **0.87** |
|  | Potto/ Angwantibo (*Perodicticus potto/ Arctocebus calabarensis*) | 0.80 | 0.68 |
|  | Putty-nosed monkey (*Cercopithecus nictitans*) | 0.66 | 0.70 |
|  | Red-eared monkey (*Cercopithecus erythrotis*) | 0.56 | 0.56 |
|  | Drill (*Mandrillus leucophaeus*) | 0.55 | 0.65 |
|  | Red-capped mangabey (*Cercocebus torquatus*) | 0.53 | 0.57 |
|  | Mona monkey (*Cercopithecus mona*) | 0.51 | 0.55 |
|  | Bushbaby (*Galago* spp.) | 0.51 | 0.47 |
|  | Red colobus (*Procolobus pennatii preussi*) | 0.23 | 0.24 |
|  | Chimpanzee (*Pan troglodytes*) | 0.15 | 0.33 |
| Testudines | Tortoise (*Kinixys sp.*) | 0.86 | 0.81 |
| Galliformes | Guinea fowl (*Guttera plumifera, Agelastes niger*) | 0.83 | 0.81 |
| Pholidota | Pangolin (*Manis* sp.) | 0.77 | 0.78 |
| Crocodilia | Crocodile (*Osteolaemus tetraspis*) | 0.72 | 0.72 |
| Bucerotiformes | Hornbill (Family: Bucerotidae) | 0.64 | 0.63 |
| Afrosoricida | Giant otter shrew (*Potamogale velox*) | 0.58 | 0.51 |
| Hyracoidea | Tree hyrax (*Dendrohyrax* *dorsalis*) | 0.54 | 0.53 |
| Musophagiformes | Great blue turaco (*Corythaeola cristata*) | 0.49 | 0.50 |
| Chiroptera | Fruit bat (Family: Pteropodidae) | 0.42 | 0.35 |
| Proboscidea | African forest elephant (*Loxodonta africana cyclotis*) | 0.03 | 0.29 |
| ^a^ Proportion of hunters who report hunting each animal/ taxa  ^b^ Proportion of participants who reported consuming each animal/taxa | | | |
