# Supplementary material for: Drivers of Bushmeat Hunting and Perceptions of Zoonoses in Nigerian Hunting Communities
Source: PLoS Negl Trop Dis. 2015 May 22;9(5):e0003792. doi: 10.1371/journal.pntd.0003792 (PMC4441483; doi:10.1371/journal.pntd.0003792)
Supplement: S3 Table — (DOCX) [file pntd.0003792.s003.docx]

| Table S3. Summary of wild animals used as traditional medicine. | | | |
| --- | --- | --- | --- |
| Animal* | Body Part | Use | # of reports |
| Monkey | skull | used as cup to drink boiled water from | 45 |
|  | feces | drank with water/ local liquor | 13 |
|  | intestine | boiled and drank broth | 2 |
|  | hair | mixed with local liquor, drank | 1 |
|  | ground bone | mixed with water, used as enema | 1 |
| Water | leg | roasted, mixed ash with water or local liquor, drank | 40 |
| chevrotain | bone | roasted, mixed ash with water, used as enema | 1 |
| Rock python | fat | Used as rub; drank with liquor; dried in sun to use as lozenge | 21 |
|  | bile | drank with liquor | 9 |
|  | kidney | put in local liquor and drank | 2 |
|  | teeth | used to lacerate boil or breast | 2 |
|  | flesh | ate | 1 |
| Blue duiker | bone | ground, mixed with water and used as rub | 26 |
|  | skull | kept palm oil in skull for rub; used as cup | 3 |
|  | flesh/ intestine | mixed with herbs, ate | 2 |
|  | roasted skin | mixed with medicine and oil, used as lozenge | 1 |
| Flying squirrel | hair or skin | used as rub | 21 |
| Red-river hog | ground bone/skull | mixed with water, used as rub | 14 |
| Tortoise | bone | tied to body | 5 |
|  | intestine/heart/skin | mixed with palm oil, drank | 3 |
|  | ground bone/shell | mixed with water, drank or used as enema; used as rub in scarifications | 3 |
| Brush-tailed | burned spines | used as rub | 3 |
| porcupine | spine | used to lacerate boil | 1 |
|  | intestine | boiled, drank broth; ate | 2 |
|  | heart | mixed with alcohol, drank | 1 |
| Monitor lizard | flesh | boiled, drank broth | 5 |
|  | skin | dried in sun, used lozenge; wore on necklace | 2 |
| African forest | feces | mixed with water, drank; used as rub | 4 |
| elephant | bile | mixed with local liquor, drank | 1 |
|  | fat | melted, drank | 1 |
|  | stomach | use for pillow, drink water from | 1 |
| Bay duiker | roasted skin | mixed with water or alcohol, drank | 4 |
|  | thigh flesh | ate | 1 |
| Potto/ | flesh/ roasted leg | ate | 3 |
| angwantibo | boiled meat | drank broth | 2 |
| Giant-pouched rat | meat/ gall bladder/ intestine | boiled, ate | 4 |
| Common genet | roasted skin | mixed with water, drank | 2 |
| African civet | scent gland | used as | 1 |
|  | roasted leg | mixed with ash and oil, drank | 1 |
| Black cobra | flesh | ate | 1 |
| Palm civet | feces | mixed with water, used as enema | 1 |
| Chimpanzee | dried finger bone | mixed with native chalk, used as rub | 1 |
| Leopard | flesh | ate | 1 |
| *Animals are listed in order of times they were mentioned as used to cure a sickness. | | | |
