# Supplementary material for: Drivers of Bushmeat Hunting and Perceptions of Zoonoses in Nigerian Hunting Communities
Source: PLoS Negl Trop Dis. 2015 May 22;9(5):e0003792. doi: 10.1371/journal.pntd.0003792 (PMC4441483; doi:10.1371/journal.pntd.0003792)
Supplement: S4 Table — (DOCX) [file pntd.0003792.s004.docx]

**Table S4. Behavioral predictors of contact with wildlife taxa**

|  |  | | | |  | | | | Estimated # ^a^  of | | | |  | | | |  | | | |
| --- | --- | --- | --- | --- | --- | --- | --- | --- | --- | --- | --- | --- | --- | --- | --- | --- | --- | --- | --- | --- |
|  | animals hunted | | | | primates hunted | | | | rodents hunted | | | | ungulates hunted | | | | carnivores hunted | | | |
| *Predictors^b^* | β | *df* | *t* | *p* | β | *df* | *t* | *p* | β | *df* | *t* | *p* | β | *df* | *t* | *p* | β | *df* | *t* | *p* |
| Constant | 12.96 | 1 | 3.44 | -- | 1.42 | 1 | 0.7 | -- | 6.89 | 1 | 5.88 | -- | -0.5 | 1 | -0.3 | -- | 8.36 | 1 | 6.42 | -- |
| Hunting frequency | 0.05 | 1 | 2.08 | <.05 | -- | -- | -- | -- | 0.03 | 1 | 3 | <.01 | 0.02 | 1 | 2.17 | <.05 | -- | -- | -- | -- |
| Sleep in forest (*frequency*) | -- | -- | -- | -- | 0 | 1 | 2.43 | <.05 | -- | -- | -- | -- | -- | -- | -- | -- | -- | -- | -- | -- |
| Hunting location | -- | -- | -- | -- | -- | -- | -- | -- | -- | -- | -- | -- | -- | -- | -- | -- | -- | -- | -- | -- |
| *Forest* | -- | -- | -- | -- | -- | -- | -- | -- | -- | -- | -- | -- | 1.97 | 1 | 2.27 | <.05 | -- | -- | -- | -- |
| *Farm* | -- | -- | -- | -- | -- | -- | -- | -- | -- | -- | -- | -- | -- | -- | -- | -- | -- | -- | -- | -- |
| *Both* | *ref* | -- | -- | -- | *ref* | -- | -- | -- | *ref* | -- | -- | -- | *ref* | -- | -- | -- | *ref* | -- | -- | -- |
| Time of day | -- | -- | -- | -- | -- | -- | -- | -- | -- | -- | -- | -- | -- | -- | -- | -- | -- | -- | -- | -- |
| *Day only* | *ref* | -- | -- | -- | *ref* | -- | -- | -- | *ref* | -- | -- | -- | *ref* | -- | -- | -- | *ref* | -- | -- | -- |
| *Night only* | 2.27 | 1 | 0.76 | *ns* | 4.97 | 1 | 2.16 | <.05 | -- | -- | -- | -- | -- | -- | -- | -- | -- | -- | -- | -- |
| *Both* | 5.57 | 1 | 2.07 | <.05 | 7.22 | 1 | 3.63 | <.001 | 1.94 | 1 | 2.49 | <.05 | -- | -- | -- | -- | -- | -- | -- | -- |
| Machete (*yes/ no*) | -- | -- | -- | -- | -- | -- | -- | -- | -- | -- | -- | -- | 2.17 | 1 | 2.26 | <.05 | -- | -- | -- | -- |
| Trap (*yes/ no*) | -- | -- | -- | -- | -- | -- | -- | -- | -- | -- | -- | -- | 3.92 | 1 | 2.21 | <.05 | -- | -- | -- | -- |
| Gun (*yes/ no*) | 3.99 | 1 | 2.31 | <.05 | -- | -- | -- | -- | 2.16 | 1 | 2.9 | <.01 | -- | -- | -- | -- | -- | -- | -- | -- |
| Dog (*yes/ no*) | 3.75 | 1 | 2 | 0.05 | 2.71 | 1 | 2.11 | <.05 | -- | -- | -- | -- | 2.54 | 1 | 3.13 | <.01 | 2.20 | 1 | 2.39 | <.05 |
| \| ^a^ Numerical response variables were transformed by square root to satisfy assumptions of normality. \|  \|  \|  \|  \|  \|  \|  \|  \|  \| \| --- \| --- \| --- \| --- \| --- \| --- \| --- \| --- \| --- \| --- \| \| ^b^ Village was incorporated as a random effect. \| \| \| \| \| \| \| \| \| \| | | | | | | | | | | | | | | | | | | | | |
