# Supplementary material for: Drivers of Bushmeat Hunting and Perceptions of Zoonoses in Nigerian Hunting Communities
Source: PLoS Negl Trop Dis. 2015 May 22;9(5):e0003792. doi: 10.1371/journal.pntd.0003792 (PMC4441483; doi:10.1371/journal.pntd.0003792)
Supplement: S1 Checklist — (DOC) [file pntd.0003792.s005.doc]

STROBE Statement—Checklist of items that should be included in reports of ***cross-sectional studies***

|  | Item No | Recommendation |
| --- | --- | --- |
| **Title and abstract** | 1 | (*a*) Indicate the study’s design with a commonly used term in the title or the abstract  “We used interview responses from hunters and non-hunters in rural hunting communities” (p2) |
|  |  | (*b*) Provide in the abstract an informative and balanced summary of what was done and what was found  “We used interview responses from hunters and non-hunters in rural hunting communities in Nigeria to: 1) quantify contact rates with wildlife, 2) identify specific hunting behaviors that increase frequency of contact, 3) identify socioeconomic factors that predispose individuals to hunt, and 4) measure perceptions of risk. Participants engaged in a variety of behaviors that increased contact with wild animals, including: butchering to sell (37%), being injured (14%), using body parts for traditional medicine (19%), collecting carcasses found in forests and/or farms (18%), and keeping as pets (16%). Hunters came into contact with wildlife significantly more than non-hunters, even through non-hunting exposure pathways. Hunters reported hunting rodents (95%), ungulates (91%), carnivores (90%), primates (78%), and bats (35%), among other prey. Reported hunting frequencies within taxonomic groups of prey were different for different hunting behaviors. Young age, lower education level, larger household size, having a father who hunts, and cultural group were all associated with becoming a hunter. Fifty-five percent of respondents were aware that they could contract diseases from wild animals, but only 26% of these individuals reported taking protective measures.” |
| Introduction | | |
| Background/rationale | 2 | “Explain the scientific background and rationale for the investigation being reported  “An estimated 282 grams of bushmeat are consumed per person per day in the Congo Basin, with over three million tons harvested in Central Africa annually [1,2] . Hunting of wild animals on this scale threatens wildlife conservation and increases risk of zoonotic disease transmission [3,4]. Rural communities across the tropical forests of West and Central Africa rely heavily on bushmeat as a nutritional, economic and cultural component of their livelihoods [5,6]. However, increasingly intense extraction is unsustainable and results in enhanced opportunities for zoonotic disease transmission [7]. A general shift towards cash economies, increased access to previously remote areas for natural resource extraction, and widespread use of guns have altered traditional hunting behavior and increased dependency on the sale of bushmeat to meet urban demands [8–12]. Market surveys in Nigeria estimate that over 900,000 kilograms of bushmeat are sold annually [13]. Large profit margins create incentives for the bushmeat trade across all levels of the supply chain, allowing bushmeat to reach national and international markets [13]. In the Ivory Coast, for example, the bushmeat trade is valued at 150 million USD [2]. An estimated five tons of bushmeat are smuggled from Africa to Europe per week [14]. Worldwide, wildlife is second only to narcotics among black market trades [15].  Frequent contact with wildlife through the bushmeat trade puts people at risk of infection with zoonotic pathogens. Pathogens transmissible to humans through bushmeat include: simian immunodeficiency virus, human T-cell lymphotrophic virus, simian foamy virus, monkeypox virus, Ebola and Marburg filoviruses, anthrax, herpes viruses, hepatitis viruses, paramyxoviruses and various parasites [16]. Among prey taxa, bats, rodents and primates consistently stand out as important sources of zoonoses. Bats and rodents have high zoonotic viral richness, and the close genetic similarity between humans and non-human primates makes exposure particularly risky[17–20]. For example, pandemic HIV originated from viruses of Central African chimpanzees, providing a striking example of the global consequences of zoonoses resulting from contact with primates [21]; and other simian retroviruses appear to “jump” between primates and people with regularity (for review see: [22]). Compared to primates, rodents are a far more abundant and geographically widespread taxon[23,24]. Forest dwelling and peridomestic rodents in West Africa host viruses such as Lassa virus and monkeypox virus, as well as a range of vector-borne pathogens [18]. Bats harbor the highest number of zoonotic viruses per host species and have received a great deal of recent attention because of outbreaks of zoonotic corona-, filo-, and paramyxoviruses [25,26]. The nature and frequency of human interaction with these and other wildlife taxa determine the pathways by which zoonotic diseases emerge.  The disruption of transmission pathways requires improved understanding of the interactions between key biological, behavioral and sociological drivers of human–animal contact. In places where reliance on wild foods and income are linked, certain individuals may be at particular risk of infection. Conventional wisdom holds that the poorest households in rural communities rely most heavily on wild foods [27–31], but this paradigm is not universal [32–34]. Still, little information exists on social and economic factors that influence whether individuals hunt. “ |
| Objectives | 3 | State specific objectives, including any prespecified hypotheses  “In this study, we conducted interviews in remote Nigerian hunting communities to identify: 1) transmission pathways by nature and frequency of interactions between humans and wildlife; and 2) socioeconomic factors that may put individuals at increased risk of zoonotic infections from wild animals. Because perceptions of risk are known to vary among hunters in West and Central Africa [30,35], we also used closed- and open-ended interviews to measure zoonotic disease awareness, perceived risk and self-protective behavior.” |
| Methods | | |
| Study design | 4 | Present key elements of study design early in the paper – “Study Design” –p.6 |
| Setting | 5 | Describe the setting, locations, and relevant dates, including periods of recruitment, exposure, follow-up, and data collection – “Study Site” Page 5 |
| Participants | 6 | (*a*) Give the eligibility criteria, and the sources and methods of selection of participants  “We enrolled participants to obtain responses from an approximately equal number of hunters and non-hunters. We first enrolled self-identified hunters and then identified non-hunters through random-selection of households. If household members chose not to participate, or were not home after three visits, we replaced the household with its nearest neighbor. During non-hunter interviews, we frequently discovered individuals who were, in fact, actively hunting or had hunted previously in their lives, and in several villages, we were unable to find a sufficient number of people who had never hunted. Because we were interested in whether or not a participant’s current social and economic situation influenced hunting behavior, we re-defined “hunter” as any individual who reported killing an animal in the past year, excluding one individual who reported killing a single snake on his farm. Enrollment was restricted to men because women in this area do not hunt.” Page 6. |
| Variables | 7 | Clearly define all outcomes, exposures, predictors, potential confounders, and effect modifiers. Give diagnostic criteria, if applicable – We designed and administered a four-part questionnaire to obtain basic demographic information, information on exposure to animals, views on the merits of hunting as a livelihood, and perceptions of zoonotic risk. Questionnaires were informed by similar studies [30,35,38], which provided the basis for establishing categories of contact modes . Local translators back-translated documents to validate the survey instrument for each village. We collected information to identify socioeconomic factors that may put individuals at risk of zoonotic infections from wild animals through hunting. These data included: age (*years*); marital status (*number of wives*); children (*number*); religion (*open*); ethnic group (*open*); education [*(0) none, (1) primary school, (2) secondary school, (3) beyond secondary school*]; primary occupations (*top 3; open*); father is/ was a hunter (*y/*n); house roof type [*(0) vegetation , (1) zinc without ceiling, (2) zinc with ceiling , (3) aluminum without ceiling, (4)* *aluminum with ceiling* ], house material [*(0) mud, (1) mud with plaster, (2) cement, (3)* *cement with plaster*], domestic animals [*animal type; (0) none, (1) 1-5, (2) 6-10 , (3) >10*]; other possessions (*generator/ television/ DVD player/ CD player/ motor bike/ cell phone*).  To assess contact frequency by species, we showed each participant published drawings of local wildlife [39] and referred to their local or English names (Table S1). For each animal, we asked participants how often they consumed, hunted, sold, received an injury from, collected if they found dead, or kept it as a pet. These behaviors are termed “risky” throughout, as they result in direct contact between humans and wildlife species. Frequency data, unless otherwise indicated, were collected on a six-level ordinal scale (*never, 1-5 times in their lifetime, 1-2 times/year, 1-2 times/ month, 1-2 times/week or daily*). The following additional data were collected from each participant: meat preference (*bushmeat/domestic meat/ top 3 preferred wild animals*), domestic and bushmeat consumption (*frequency*), whether they: butchered bushmeat to sell (*y/n/average price*); accidently cut themselves while butchering (*y/n*); used bushmeat for medicinal purposes (*y/n; animal type; description of use*); adhered to local taboos or laws against killing and/or consuming wild animals (*y/n; examples*); and used bushmeat for cultural purposes (*y/n; examples*). The following information was collected from hunters only: easiest animals to hunt (*top 3; open*), most desirable prey (*top 3; open*); hunting technique (*gun/ trap/ machete/dog*), hunting location (*forest/farm/both*), hunting time (*night/day/both*), hunting season (*occasional/wet/dry/all year*), hunting frequency, and whether they slept in the forest while on hunting excursions (*frequency*).  To characterize individual views of participants on the merits of hunting as a livelihood, we asked hunters whether they would still hunt if they had alternatives (*y/n/sometimes),* and if they wanted their children to hunt (*y/n; why or why not*). Finally, to measure zoonotic disease awareness, perceived risk, and precautions taken to mitigate exposure, we collected data on knowledge of wildlife zoonosis (*y/n; types of diseases; source animals*); source of information (*open*); perceived threat (*y/n*); and precautionary measures taken (*y/n; explain*). - pages 7-8 |
| Data sources/ measurement | 8* | For each variable of interest, give sources of data and details of methods of assessment (measurement). Describe comparability of assessment methods if there is more than one group - “**Analyses**  We used data on roofing material, housing material and household assets to create an index of household wealth. This index was based on published results of participatory and small-scale survey research comparing livelihood data for a range of households relying on non-timber forest goods in West and Central Africa [40]. Specifically, we assigned points based on roofing material (0-4), housing material (0-3), number of livestock (0-3), and non-essential household items (0-6). Thus the maximum possible score was sixteen; the higher the score, the wealthier the household.  For certain analyses we converted hunting and consumption frequencies from ordinal to conservative numeric estimates of minimum yearly off-take, in units of numbers of animals (never = 0, rarely = 0, yearly = 1, monthly = 12, weekly = 52 and daily = 104) for incorporation into generalized linear models. For less frequent behaviors (collect dead, injured by, kept as pet), we used the total number of animals contacted over the participant’s lifetime.  For hunters who reported hunting daily, weekly, or monthly but only during one season, we corrected hunting frequency estimates by one-half (seasonal hunters) or one-third (occasional hunters). To determine whether there were significant differences in reported contact between hunters and non-hunters, we used chi-square tests. For modes of contact where most participants engaged in the specific behavior, we compared ordinal frequencies of contact using Mann-Whitney U tests. We constructed generalized linear mixed models to examine behavioral and socioeconomic predictors of individual hunting activity and frequency of contact with wildlife. Since modes of contact are not mutually exclusive (e.g. animals can be hunted and sold or hunted and consumed), and covariance among these factors makes it difficult to separate the effect of any single behaviors on overall risk, we limited our analyses of behavioral and socio-economic predictors of risk to hunting behavior alone.  To identify hunting behaviors significantly associated with high frequency of contact with all wild animals, and with specific taxa, we used mixed effects linear regression models with backwards elimination of behavioral predictor variables. We then used the same selection method in a mixed effects logistic regression model to determine which socioeconomic variables were significantly associated with being a hunter. We incorporated village as a random effect in all models. We performed analyses with *nlme* and *glmer* functions in RGui (3.0.2)[41]. We initially included all variables in the models; however, we retained only significant variables (at the alpha=.05 level) and first-order interactions among significant main effects in the final model.” -pages 9-10 |
| Bias | 9 | Describe any efforts to address potential sources of bias  “We enrolled participants to obtain responses from an approximately equal number of hunters and non-hunters. We first enrolled self-identified hunters and then identified non-hunters through random-selection of households.” - p6 |
| Study size | 10 | Explain how the study size was arrived at  “We enrolled participants to obtain responses from an approximately equal number of hunters and non-hunters.” - p6  “To increase the generality of our results, we selected communities that varied in proximity to the national park (outside, support zone, or enclave) and cultural group (primarily Efik or Ejagham).” – p5  Sample sizes were determined by the number of hunters in each village. |
| Quantitative variables | 11 | Explain how quantitative variables were handled in the analyses. If applicable, describe which groupings were chosen and why  “**Analyses**  We used data on roofing material, housing material and household assets to create an index of household wealth. This index was based on published results of participatory and small-scale survey research comparing livelihood data for a range of households relying on non-timber forest goods in West and Central Africa [40]. Specifically, we assigned points based on roofing material (0-4), housing material (0-3), number of livestock (0-3), and non-essential household items (0-6). Thus the maximum possible score was sixteen; the higher the score, the wealthier the household.  For certain analyses we converted hunting and consumption frequencies from ordinal to conservative numeric estimates of minimum yearly off-take, in units of numbers of animals (never = 0, rarely = 0, yearly = 1, monthly = 12, weekly = 52 and daily = 104) for incorporation into generalized linear models. For less frequent behaviors (collect dead, injured by, kept as pet), we used the total number of animals contacted over the participant’s lifetime.  For hunters who reported hunting daily, weekly, or monthly but only during one season, we corrected hunting frequency estimates by one-half (seasonal hunters) or one-third (occasional hunters).” |
| Statistical methods | 12 | (*a*) Describe all statistical methods, including those used to control for confounding  “To determine whether there were significant differences in reported contact between hunters and non-hunters, we used chi-square tests. For modes of contact where most participants engaged in the specific behavior, we compared ordinal frequencies of contact using Mann-Whitney U tests. We constructed generalized linear mixed models to examine behavioral and socioeconomic predictors of individual hunting activity and frequency of contact with wildlife. Since modes of contact are not mutually exclusive (e.g. animals can be hunted and sold or hunted and consumed), and covariance among these factors makes it difficult to separate the effect of any single behaviors on overall risk, we limited our analyses of behavioral and socio-economic predictors of risk to hunting behavior alone.  To identify hunting behaviors significantly associated with high frequency of contact with all wild animals, and with specific taxa, we used mixed effects linear regression models with backwards elimination of behavioral predictor variables. We then used the same selection method in a mixed effects logistic regression model to determine which socioeconomic variables were significantly associated with being a hunter. We incorporated village as a random effect in all models. We performed analyses with *nlme* and *glmer* functions in RGui (3.0.2)[41]. We initially included all variables in the models; however, we retained only significant variables (at the alpha=.05 level) and first-order interactions among significant main effects in the final model.” |
|  |  | (*b*) Describe any methods used to examine subgroups and interactions  We initially included all variables in the models; however, we retained only significant variables (at the alpha=.05 level) and first-order interactions among significant main effects in the final model.” |
|  |  | (*c*) Explain how missing data were addressed  “We omitted cases with missing values from our analyses.” P. 9 |
|  |  | (*d*) If applicable, describe analytical methods taking account of sampling strategy |
|  |  | (*e*) Describe any sensitivity analyses |
| Results | | |
| Participants | 13* | (a) Report numbers of individuals at each stage of study—eg numbers potentially eligible, examined for eligibility, confirmed eligible, included in the study, completing follow-up, and analysed  “We interviewed 327 participants”  “We enrolled participants to obtain responses from an approximately equal number of hunters and non-hunters. We first enrolled self-identified hunters and then identified non-hunters through random-selection of households. If household members chose not to participate, or were not home after three visits, we replaced the household with its nearest neighbor. During non-hunter interviews, we frequently discovered individuals who were, in fact, actively hunting or had hunted previously in their lives, and in several villages, we were unable to find a sufficient number of people who had never hunted. Because we were interested in whether or not a participant’s current social and economic situation influenced hunting behavior, we re-defined “hunter” as any individual who reported killing an animal in the past year, excluding one individual who reported killing a single snake on his farm. Enrollment was restricted to men because women in this area do not hunt.” P.6 |
|  |  | (b) Give reasons for non-participation at each stage n/a |
|  |  | (c) Consider use of a flow diagram |
| Descriptive data | 14* | (a) Give characteristics of study participants (eg demographic, clinical, social) and information on exposures and potential confounders  “Demographic information was collected from 327 individuals, representing 188 hunters and 139 non-hunters. The median age of all participants was 31.5 (range = 15-93) years. Fifty percent of individuals had the equivalent of a primary school education or lower, 32% had finished secondary school, and 18% had at least one year of higher education. Sixty-nine percent of individuals were married, and 7% had multiple wives. The average number of children was four (range = 0-26). The study populations were predominantly Christian (93%), with the remainder practicing traditional religions (6%) or Islam (1%). Participants identified their tribal affiliations primarily as Efik (73%), Ejagham (14%), and a variety of other (primarily Ibibio from neighboring Akwa Ibom state) cultural groups (13%). Farming (subsistence agricultural and selling of crops) was the most common occupation (69%). Hunting (33%) and trapping (19%) were the second and third most common occupations, followed by salaried work (15%), being in school (12%), having a skilled trade (10%), selling goods (7%), driving a motorbike taxi (5%), being a village leader, being unemployed, collecting forest goods, being a member of the clergy, collecting palm wine, fishing, and livestock farming (each less than 5%).” P9-10 |
|  |  | (b) Indicate number of participants with missing data for each variable of interest  **Demographic Information**  “Demographic information was collected from 327 individuals, representing 188 hunters and 139 non-hunters.”  Sample sizes provided for all variables with missing data:  “Fifty percent (n=163/323) of individuals had the equivalent of a primary school education or lower, 32% had finished secondary school, and 18% had at least one year of higher education. Sixty-nine percent (n=223/325) of individuals were married, and 7% had multiple wives. The average number of children was four (range = 0-26). The study populations were predominantly Christian (93%, n=302/324), with the remainder practicing traditional religions (6%) or Islam (1%). Participants identified their tribal affiliations primarily as Efik (73%), Ejagham (14%), and a variety of other (primarily Ibibio from neighboring Akwa Ibom state) cultural groups (13%).”  **Reasons for Hunting**  “ Seventy-five percent (n=111/156) of hunters had fathers who were also hunters.  Eighty-four percent (n=145/173) of participants reported that they would choose not to hunt if they had an alternative source of income. Ninety-seven percent of participants reported not wanting their children to hunt. The most common reason that people gave for not wanting their children to hunt was that hunting was too difficult (49%; n=159/324).” |
| Outcome data | 15* | Report numbers of outcome events or summary measures |
| Main results | 16 | (*a*) Give unadjusted estimates and, if applicable, confounder-adjusted estimates and their precision (eg, 95% confidence interval). Make clear which confounders were adjusted for and why they were included  “We incorporated village as a random effect in all models. We performed analyses with *nlme* and *glmer* functions in RGui (3.0.2)[41].” P.10  See table 2  See table S4 |
|  |  | (*b*) Report category boundaries when continuous variables were categorized  “For certain analyses we converted hunting and consumption frequencies from ordinal to conservative numeric estimates of minimum yearly off-take, in units of numbers of animals (never = 0, rarely = 0, yearly = 1, monthly = 12, weekly = 52 and daily = 104) for incorporation into generalized linear models. For less frequent behaviors (collect dead, injured by, kept as pet), we used the total number of animals contacted over the participant’s lifetime. We omitted cases with missing values from our analyses.  For hunters who reported hunting daily, weekly, or monthly but only during one season, we corrected hunting frequency estimates by one-half (seasonal hunters) or one-third (occasional hunters).” P.9 |
|  |  | (*c*) If relevant, consider translating estimates of relative risk into absolute risk for a meaningful time period - n/a |
| Other analyses | 17 | Report other analyses done—eg analyses of subgroups and interactions, and sensitivity analyses – “We initially included all variables in the models; however, we retained only significant variables (at the alpha=.05 level) and first-order interactions among significant main effects in the final model.”- P.10 |
| Discussion | | |
| Key results | 18 | Summarise key results with reference to study objectives  “We found that younger age, lower education level, larger household size, having a father who hunts, and being of the resident cultural group were all significantly associated with becoming a hunter. Hunters had more frequent contact with wildlife through both hunting and non-hunting behaviors, likely experiencing higher exposure risk to zoonosis than non-hunters. Specific hunting behaviors, namely high hunting frequency, hunting during both day and night, hunting specifically at night, and hunting with a gun and with a dog were all associated with high rates of contact with wildlife. Other behaviors were associated with higher rates of contact with specific taxa, namely: sleeping in the forest (primates), and using a machete and trap (ungulates). Carnivore and rodent hunting frequency was not uniquely associated with any specific hunting behaviors  Our results shed new light on the social-cultural contexts of wildlife contact in this region and have implications for conservation and public health.” |
| Limitations | 19 | Discuss limitations of the study, taking into account sources of potential bias or imprecision. Discuss both direction and magnitude of any potential bias  “ Since modes of contact are not mutually exclusive (e.g. animals can be hunted and sold or hunted and consumed), and covariance among these factors makes it difficult to separate the effect of any single behaviors on overall risk, we limited our analyses of behavioral and socio-economic predictors of risk to hunting behavior alone.” |
| Interpretation | 20 | Give a cautious overall interpretation of results considering objectives, limitations, multiplicity of analyses, results from similar studies, and other relevant evidence  “We found a negative association between education level and hunting, and no effect of wealth. These results differ from those of Le Breton and colleagues (2006) who found that hunting in Cameroon was more common among poorer households (as measured by roof type) with no effect of education level*.* Similarly, in Tanzania, participation in illegal hunting decreased with increasing wealth, as measured by ownership of sheep and goats [28]. Negative results in our study may reflect low variation in economic status, in that all participants were almost uniformly materially disadvantaged. This conclusion is supported by our observation that larger family sizes appeared to generate a greater need for income, which may be most accessible through hunting, particularly for individuals from families with experienced hunters. Individuals with higher education levels, a factor associated with lower probability of hunting in our study, do not necessarily have higher income, but may engage in activities that generate extra income or in other commitments that keep them out of the forest.  Our data also show that resident cultural groups were more likely to hunt than other cultural groups, which tend to be migrants from nearby states. Our study sites varied in numbers of migrants from neighboring states, but when present, they commonly resided in the periphery of villages as farmers and were not permitted to hunt by order of village chiefs. Our results contrast those of other studies that found that migrants hunted a majority of bushmeat (Congo), had higher rates of primate contact (Uganda), and were more likely to be involved in butchering (Cameroon) than resident groups [12,30,42]. Our results may reflect cultural differences among migrant populations, or a unique local response from resident cultural groups who fear loss of livelihoods to migrant populations.  Although hunting is illegal and considered an undesirable livelihood, strong incentives to hunt still persist. Indeed, we struggled to identify men who had never hunted or trapped wildlife. Nevertheless, almost all participants claimed that if given an alternative, they would choose not to hunt. Virtually all said they did not want their children to become hunters because it is too difficult, dangerous, stressful, not a legitimate occupation, and is no longer profitable. This contrasts directly with historical accounts of hunting in this region, in which hunters were described as being “economically independent” and “far too important a person” to employ [46; pg.152] . We suggest that declines in wildlife numbers coupled with increasing distances between wildlife habitat and villages have decreased incentives to hunt. With lower returns per hunt, those who are able turn to alternatives. Many who continue to hunt do so out of necessity, and in turn, hunting is viewed as a low-merit livelihood, even among hunters themselves. While preference for bushmeat will inevitably drive the trade to a certain degree, our data suggest that provision of alternative livelihoods would reduce hunting behavior by restricting hunting frequency and providing supplemental income. However, individuals in need of extra income would remain free to hunt at night and set traps, which were predictors of primate and ungulate hunting frequency, respectively.  Nearly all participants reported consuming bushmeat, and there was a strong preference for bushmeat over domestic meat. A majority of participants had strong cultural ties to the consumption of bushmeat, and very few recognized laws protecting wildlife. A majority of hunters reported selling bushmeat, indicating that demand from rural and urban markets continue to provide incentives for bushmeat hunters, who often lack alternative ways to generate income. These incentives may be modified by hunting taboos. For example, in neighboring states in Nigeria, certain guenons are held as deities and are protected within particular villages [44]. In this region however, such taboos were uncommon. As a result, hunting in and near protected areas remains common in Nigeria [13,45].  Although previous studies reported frequencies of carcasses in bushmeat markets in this region [13], we are aware of none examining hunting preferences and cultural uses that may be driving human-wildlife contact at the local level. Significantly, we found that participants reported having had contact with primates more than with any other wildlife taxon. This remains true whether keeping wildlife as pets is included in our analyses, since it was reported far less frequently than hunting. However, we note that keeping animals as pets presents a different kind of risk, in which people come into frequent contact with animals over a prolonged period. This behavior, unlike others, leads to opportunities for repeated injury and exposure to animals that may be persistently stressed.  Eighty-seven percent of individuals reported consuming primates, and monkeys were listed among the most desirable animals to eat and were most frequently mentioned as useful for medicinal purposes or kept as a pet. These data parallel high primate consumption rates [30], and preferences for primates [35] documented for other regions. Porcupine and blue duiker were consumed by over 90% of individuals, with porcupine most frequently mentioned as a preferred meat. Ebola epidemics have been previously associated with handling duiker carcasses [46], and though we are unaware of zoonotic viruses transmitted directly through contact with porcupines, rodents in general host more than 60 known zoonotic viruses. Bats, along with other small prey, were anecdotally referred to as “children’s meat”, in that they are small and thus given to children to play with and eat, thereby potentially putting children at greater risk. The link between bushmeat hunting and zoonotic disease risk through such pathways has been discussed extensively[17,18,30]; our data expand these risks to a new region and a new cultural setting.  Of the 55% of participants who reported awareness of zoonotic diseases from wildlife, a majority reported believing that there was an actual risk associated with contact. Awareness of wildlife zoonoses was considerably higher than reported in hunting communities in Sierra Leone ([35]; 55% *versus* 24%), but overall perceived risk was lower than in Cameroon ([30]; 46% *versus* 74%). Differences across study sites may be due to educational campaigns in the respective areas. We are unaware of public health outreach campaigns related to wildlife and disease in this region. However, given the proximity to the national park, participants may have previously received information of risks associated with hunting and consumption of wildlife species of conservation concern, particularly primates. In our study, information about risk came primarily through broadcast news outlets, forestry/conservation personnel, and word of mouth. Only one individual reported a public health official as a source of information about zoonotic diseases, despite the fact that such individuals are in strong positions to enhance knowledge of risks associated with bushmeat, especially near protect areas where wildlife contact rates are high.  Fifty-five percent of participants who reported awareness of wildlife zoonoses gave monkeys and HIV as an example. However, many other examples were of unconfirmed hosts or non-zoonotic pathogens. Despite such knowledge, very few individuals reported protecting themselves from infection. Avoidance was the most frequently cited protective measure, including avoidance of eating bushmeat, touching blood, sexual contact, or eating fruit from trees where monkeys had been feeding. Of those who protected themselves, 31% reported taking traditional and/or commercial medicine as a treatment or prophylaxis. Many potential zoonoses are viral and therefore locally available treatments such as saline injections, antibiotics and acetaminophen would be ineffective. Additionally, the effectiveness of traditional treatments such as consumption of wild herbs or bitter cola (*Garcinia afzelii*) against zoonotic pathogens is as yet unproven. Only five percent of participants reported using safe meat handling practices, such as cleaning or cooking meat well prior to consumption, as a protective measure. We recorded differences in consumption patterns among locations (e.g. consumption of partially smoked innards at the hunting sheds *versus* well-smoked meat sold in markets), suggesting that risk of contact and zoonoses varies across space and time. Participants also reported wearing clothes and/or boots for protection, for example when hunters carry carcasses over long distances (wearing clothing) or restrain animals with their feet (wearing boots). One participant reported wearing protective gloves while butchering. The efficacy of these measures for protecting against exposure to infectious material is unknown, but is likely to be higher than using no protection at all. Education programs implemented through conservation programs and/or news outlets should therefore include information on avoidance strategies, with specific attention to dispelling misconceptions about routes of transmission and promoting effective and accessible strategies for mitigating exposure.” |
| Generalisability | 21 | Discuss the generalisability (external validity) of the study results  “Our findings highlight the value of understanding socio-cultural drivers of bushmeat hunting for reducing contact with wildlife in high-risk groups. Hunting wildlife for meat is widespread in West and Central Africa, and effective public health solutions are unlikely to emerge from conservation and regulatory agencies alone. Our data suggest that effective solutions will include implementation of alternative livelihood programs specifically targeting hunters and aimed at providing alternative protein sources that would satisfy local taste preferences (e.g. raising desirable species in captivity [47,48]). Conservation rules that limit hunting, or prohibit hunting with dogs, and are implemented with the help of local chiefs may be most effective in reducing hunting pressure, such as in the case of effectively restricting hunting privileges to resident groups. However, given the cultural and economic contexts of the bushmeat trade, a complete shift to alternative protein sources may be impractical at present.  Novel self-protective strategies should be developed through consultation with individuals who currently protect themselves, make use of locally available goods, and be tested locally for cultural acceptability. Our data suggest that conservation and public health initiatives that tap into existing outlets for transmitting information, such as word of mouth and radio broadcasting, are likely to be most effective in reaching and influencing people in high risk areas. Although our study focuses on drivers of hunting, a behavior practiced only by men in this region, women are at risk from butchering and trading the animals brought back by hunters [35,49], and should also be targeted during educational programs and interventions.  Results from Nigeria demonstrate that hunters in this setting frequently contact a diversity of prey in “risky” ways, and that the decision to become a hunter is rooted in family tradition, modified by economic necessity. Improved education, reduced family sizes, and provision of alternative livelihoods may result in reduced contact with wildlife and lower zoonotic disease risk in rural hunting communities in Nigeria and similar locations. We acknowledge that such solutions require the mobilization of significant resources toward development and conservation jointly. We also advocate targeting neglected transmission pathways, such as distinct cultural uses of wildlife that provide novel routes for pathogen exchange. These potential routes of transmission have received less attention than those associated with hunting of bushmeat for consumption, but they may in aggregate confer equal or greater risk.” |
| Other information | | |
| Funding | 22 | Give the source of funding and the role of the funders for the present study and, if applicable, for the original study on which the present article is based  See funding information:  “Fulbright Institute of International Educational”  “National Institutes of Health - T32AI007414” |

*Give information separately for exposed and unexposed groups.

**Note:** An Explanation and Elaboration article discusses each checklist item and gives methodological background and published examples of transparent reporting. The STROBE checklist is best used in conjunction with this article (freely available on the Web sites of PLoS Medicine at http://www.plosmedicine.org/, Annals of Internal Medicine at http://www.annals.org/, and Epidemiology at http://www.epidem.com/). Information on the STROBE Initiative is available at www.strobe-statement.org.
